# EGFL7 loss correlates with increased VEGF-D expression, upregulating hippocampal adult neurogenesis and improving spatial learning and memory

**DOI:** 10.1007/s00018-023-04685-z

**Published:** 2023-01-30

**Authors:** Kathrin Barth, Verica Vasić, Brennan McDonald, Nora Heinig, Marc-Christoph Wagner, Ulrike Schumann, Cora Röhlecke, Frank Bicker, Lana Schumann, Konstantin Radyushkin, Jan Baumgart, Stefan Tenzer, Frauke Zipp, Matthias Meinhardt, Kari Alitalo, Irmgard Tegeder, Mirko H. H. Schmidt

**Affiliations:** 1 Institute of Anatomy, Medical Faculty Carl Gustav Carus, Technische Universität Dresden School of Medicine, Fetscherstr. 74, 01307 Dresden, Germany; 2 Institute of Anatomy, University Medical Center of the Johannes Gutenberg University Mainz, Mainz, Germany; 3 Focus Program Translational Neuroscience (FTN), University Medical Center of the Johannes Gutenberg University Mainz, Mainz, Germany; 4 Institute of Medical Informatics and Biometry, Medical Faculty Carl Gustav Carus, Technische Universität Dresden School of Medicine, Dresden, Germany; 5 Institute of Clinical Pharmacology, Goethe-University Hospital Frankfurt Am Main, Frankfurt, Germany; 6 Institute of Pathology, University Hospital Carl Gustav Carus, Technische Universität Dresden, Dresden, Germany; 7 Mouse Behavior Outcome Unit, Johannes Gutenberg University Mainz, Mainz, Germany; 8 Translational Animal Research Center (TARC), University Medical Center of the Johannes Gutenberg University Mainz, Mainz, Germany; 9 Institute of Immunology, University Medical Center of the Johannes Gutenberg University Mainz, Mainz, Germany; 10 Focus Program Immunotherapy (FZI), University Medical Center of the Johannes Gutenberg University Mainz, Mainz, Germany; 11 Department of Neurology, Rhine-Main Neuroscience Network (rmn2), University Medical Center of the Johannes Gutenberg University Mainz, Mainz, Germany; 12 Translational Cancer Medicine Program and iCAN Digital Precision Cancer Medicine Flagship, Faculty of Medicine, University of Helsinki, Helsinki, Finland

**Keywords:** Subgranular zone, Hippocampus, Neural stem cell, Adult neurogenesis

## Abstract

Neural stem cells reside in the subgranular zone, a specialized neurogenic niche of the hippocampus. Throughout adulthood, these cells give rise to neurons in the dentate gyrus, playing an important role in learning and memory. Given that these core cognitive processes are disrupted in numerous disease states, understanding the underlying mechanisms of neural stem cell proliferation in the subgranular zone is of direct practical interest. Here, we report that mature neurons, neural stem cells and neural precursor cells each secrete the neurovascular protein epidermal growth factor-like protein 7 (EGFL7) to shape this hippocampal niche. We further demonstrate that EGFL7 knock-out in a Nestin-CreERT2-based mouse model produces a pronounced upregulation of neurogenesis within the subgranular zone. RNA sequencing identified that the increased expression of the cytokine VEGF-D correlates significantly with the ablation of EGFL7. We substantiate this finding with intraventricular infusion of VEGF-D upregulating neurogenesis in vivo and further show that VEGF-D knock-out produces a downregulation of neurogenesis. Finally, behavioral studies in EGFL7 knock-out mice demonstrate greater maintenance of spatial memory and improved memory consolidation in the hippocampus by modulation of pattern separation. Taken together, our findings demonstrate that both EGFL7 and VEGF-D affect neurogenesis in the adult hippocampus, with the ablation of EGFL7 upregulating neurogenesis, increasing spatial learning and memory, and correlating with increased VEGF-D expression.

**Supplementary Information:**

The online version contains supplementary material available at 10.1007/s00018-023-04685-z.

## Introduction

Adult neurogenesis involves the de novo formation of functional neurons from neural stem cells (NSCs) in restricted regions of the mammalian brain. Substantiated in various species [1–4], including humans [5–9], the lifetime generation and maturation of neurons in adults is believed to contribute to both the maintenance and plasticity of select neural networks. In rodents, adult neurogenesis occurs in two localized regions: i) the subventricular zone (SVZ) of the lateral ventricles and ii) the subgranular zone (SGZ) of the dentate gyrus (DG) of the hippocampus [10]. Indeed, this latter region is of particular interest, as the hippocampus is crucially involved in learning, memory and spatial navigation and is often significantly disrupted in neurological and psychiatric disease states [11]. Despite the total number of neurons in the DG being significantly larger than that of adult-born neurons, these newly created cells are able to integrate into established networks of the hippocampus and thus offer a unique form of neuronal plasticity [8, 12–14]. Therefore, insights into the molecular mechanisms underlying adult neurogenesis in the SGZ are of particular importance in advancing our understanding of hippocampal contributions to cognitive function across the lifespan.

In the hippocampus, adult neurogenesis is regulated by biochemical and physiological factors [15], as well as environmental cues and experience [16]. In particular, the Notch signaling pathway is a key regulator of NSC maturation and neuron formation [17] during embryological [18] and adult neurogenesis [19]. Specifically, Notch signaling regulates both the proliferation and cell fate decisions of NSCs and intermediate progenitor cells [20]. In animal models, conditional knock-out (KO) of Notch in NSCs, as well as depletion of the Notch-activated transcription factor, RBP-J, results in reduction in the number of NSCs, intermediate progenitor cells, and adult-born neurons [20, 21]. The activation of Notch receptors typically occurs in response to canonical Notch ligands of the Delta- and Jagged-type [22]. However, we have previously demonstrated modulation of this interface in the SVZ by the non-canonical Notch ligand epidermal growth factor-like protein 7 (EGFL7). Expression of EGFL7 increases in cells of the maturing brain until young adulthood and remains at high levels until old age [23]. In the SVZ, EGFL7 is produced in substantial quantities by NSCs but it is also secreted by neurons and endothelial cells [24] throughout the adult brain [25, 26]. Previously, we were able to show that EGFL7 antagonizes Jagged1-induced Notch signaling [27] while stimulating Dll4-induced [28] activation of Notch receptors in NSCs. In doing so, EGFL7 governed adult neurogenesis and network activity in the olfactory bulb. In particular, we observed severe deficits in olfaction upon EGFL7 KO in mice.

However, despite the importance of EGFL7 in adult neurogenesis, the protein’s expression and influence in the hippocampus has yet to be characterized, and its potential impact on learning and memory remains unclear. Therefore, our goal was to investigate if EGFL7 is involved in adult neurogenesis in the SGZ of the DG. Based on our previous findings in the SVZ, and assuming that the expression and influence of EGFL7 will be consistent across both neurogenic niches, we hypothesized the following: The non-canonical Notch ligand EGFL7 will be expressed in the hippocampus and will influence SGZ neurogenesis via modulation of Notch signaling pathways. Additionally, due to the previously observed hyposmia in EGFL7 KO mice, we hypothesized these mice would also show a disruption in cognitive functions associated with the DG.

In the current study, we demonstrate the location of EGFL7 expression in the hippocampus of mice and humans. We further report an upregulation of SGZ neurogenesis in the absence of EGFL7 and show that, in contrast to our hypothesis, the expression of the cytokine VEGF-D correlates significantly with this process, while Notch signaling is most likely not involved. We then independently verify the influence of VEGF-D, demonstrating that interventricular infusion of VEGF-D upregulates neurogenesis, while ablation of VEGF-D produces a decrease in neurogenesis in the SGZ. Finally, we illustrate increased behavioral measures of learning and memory consolidation in EGFL7 KO mice as compared to wild-type (WT) littermates.

## Results

### EGFL7 is expressed in the hippocampus of mice and humans

**Fig. 1:**
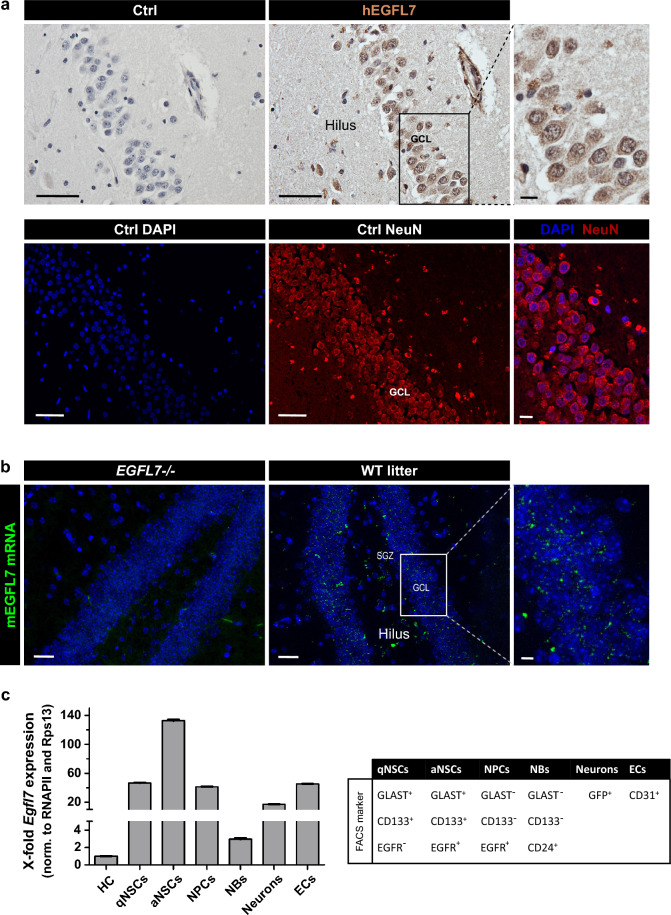
Localization of EGFL7 in the human and mouse dentate gyrus. **a** Immunoperoxidase staining revealed EGFL7 expression in granular cells (NeuN +) and larger blood vessels of the human dentate gyrus as well as larger neurons of the hilus. Scale bars represent 50 µm or 10 µm (magnification). **b** Furthermore, EGFL7 was expressed in the dentate gyrus of mice as detected by fluorescence in situ hybridization (FISH). Scale bars represent 50 µm or 10 µm (magnification). **c** Cells of murine hippocampi were isolated by fluorescence-activated cell sorting using a combination of the following markers: GFAP^+^/CD133^+^/EGFR^−^ for quiescent neural stem cells (qNSCs), GFAP^+^/CD133^+^/EGFR^+^ for active NSCs (aNSC), GLAST^−^/CD133^−^/EGFR^+^ for neural progenitor cells (NPCs), CD24^+^ for neuroblasts (NBs), Thy-GFP1^+^ for neurons and CD31^+^ for endothelial cells (ECs). Expression of *Egfl7* was measured by quantitative reverse transcriptase-polymerase chain reaction using two housekeeping genes and data were plotted as normalized to unsorted hippocampus tissue (HC). Data are presented as mean values with 95% confidence interval (CI)

To visualize the expression and localization of EGFL7 in the hippocampus, we analyzed human specimens by immunoperoxidase staining and mouse specimens by fluorescence in situ hybridization (FISH). Human hippocampus sections displayed a strong positive staining for EGFL7 in neurons of the DG and the hilus (Fig. 1a). Additionally, we performed a differential gene expression analysis showing that EGFL7 expression in the murine hippocampus is significantly lower compared to human samples (p-value = 1.157e-06). In absolute values we observe a two-fold increase (log2FC = 0.956 ± 0.190) in EGFL7 expression in human samples (Supplementary Fig. 1).

In adult mice, FISH revealed a significant expression of EGFL7 throughout the DG. We detected no signal in the DG of *EGFL7*-/- mice, thus verifying probe specificity (Fig. 1b). To identify the cellular origins of EGFL7 in the hippocampus, tissues were minced and cells of the DG separated by fluorescence-activated cell sorting. Sorted cells were analyzed by quantitative reverse transcriptase-polymerase chain reaction (qRT-PCR), which revealed a ubiquitous transcription of *Egfl7* with substantial differences in transcript abundance between cell types (Fig. 1c). High levels of *Egfl7* were synthesized in neurons (17.2 ± 0.16-fold enriched as compared to unsorted hippocampal tissue), NPCs (41.4 ± 0.21-fold), quiescent NSCs (qNSCs; 46.7 ± 0.37-fold) and activated NSCs (aNSCs; 132.7 ± 1.50-fold). These latter cells displayed the highest expression levels with close to a threefold increase as compared to qNSCs. Smaller amounts of *Egfl7* were detected in neuroblasts (NBs; 3.0 ± 0.11-fold increase). Taken together, our results demonstrate strong expression of EGFL7 in neurons, NSCs, and NPCs in the DG of the hippocampus.

### Loss-of-EGFL7 promotes the sustained proliferation of NSCs

**Fig. 2:**
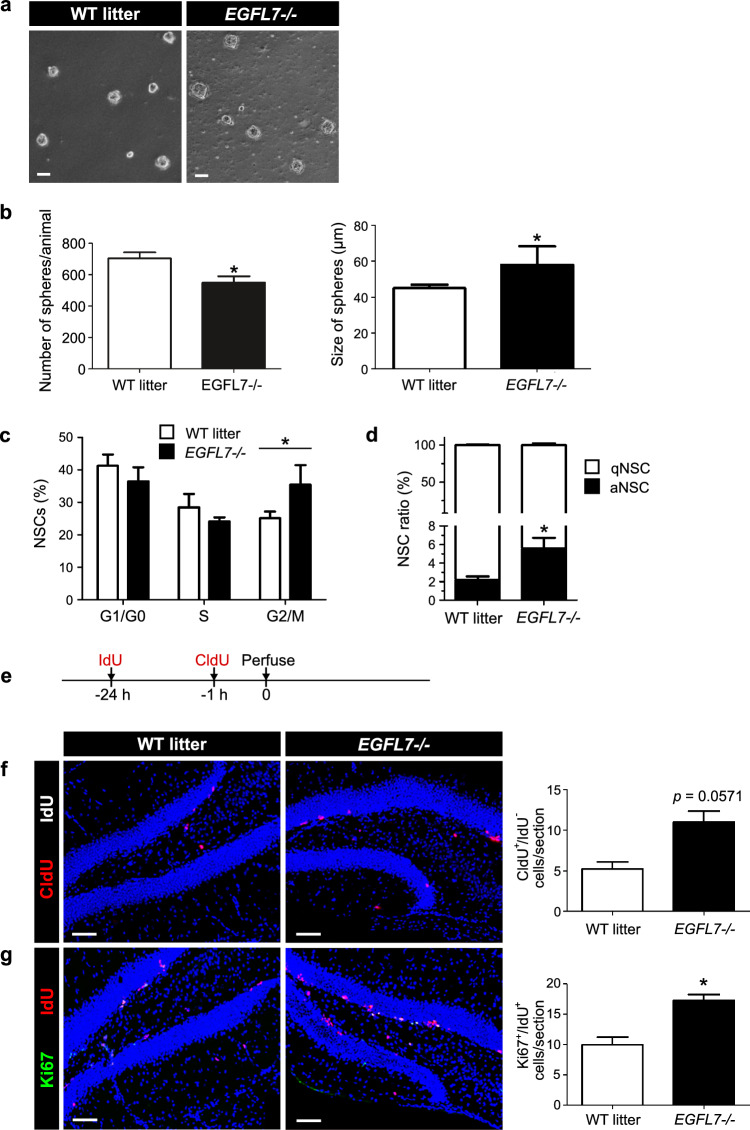
EGFL7 governs proliferation of neural stem cells in the hippocampus. **a** Representative images of neural stem and progenitor cells isolated from mouse hippocampus (HC) and cultured as spheres. Scale bars represent 25 µm. **b** Size of *EGFL7*-/- neurospheres was increased (58.95 ± 11.24 µm (*n* = 5) versus 43.85 ± 2.25 µm in WT litters; *n* = 4; *p* = 0.0317). The number of spheres derived from *EGFL7*-/- HC was decreased in the neurosphere assay. **c** Fluorescence-activated cell sorting revealed an increase in activated neural stem cells (aNSCs) in *EGFL7*-/- mice (5.56 ± 1.65% versus 2.20 ± 0.53% in WT litters, *n* = 3; *p* = 0.0499) but about equal amounts of quiescent qNSCs. **d** Flow cytometry-based cell cycle analysis of *EGFL7*-/- and WT neurospheres yielded an increased amount of cells in the G2/M phase in *EGFL7*-/- mice (33.65 ± 6.10% versus 25.10 ± 2.03%; *n* = 3; *p* = 0.0286). **f** Representative images of the dentate gyrus 24 h post administration of IdU and double-stained for Ki67/IdU. Quantification revealed sustained proliferation in *EGFL7*-/- mice (17.25 ± 2.06 versus 10.00 ± 2.45 cells per section in WT; *n* = 4; *p* = 0.0286). Scale bars represent 90 µm. Statistical analysis was performed by Mann–Whitney *U*-test. Data are represented as mean ± SEM; *p < 0.05

The functional impact of EGFL7 on NSCs/NPCs in vitro was assessed in hippocampus-derived neurospheres of *EGFL7-/-* mice as compared to WT littermates. Spheres of *EGFL7-/-* mice grew larger in diameter (58.95 ± 11.24 µm versus 43.85 ± 2.25 µm in WT litters; Fig. 2a, b), suggesting an increased proliferation rate of aNSCs/NPCs upon EGFL7 KO, which may be due to increased NSC activation or sustained proliferation. Additionally, the number of spheres was slightly reduced in the EGFL7 KO indicating less NSCs compared to WT litters (Fig. 2b). A cell cycle analysis of isolated NSCs revealed that the deletion of EGFL7 diminished the amount of cells in G1 phase, but led to a concomitant increase of cells in G2/M phase (Fig. 2c), pointing towards an increased activation rate of qNSCs. To test this hypothesis, we subjected NSCs isolated from the hippocampus of *EGFL7*-/- and WT litters to flow cytometry analyses using three cellular markers (GLAST^+^/CD133^+^/EGFR^−/+^) to discriminate between qNSCs and aNSCs (Supplementary Fig. 2a). RNA sequencing of sorted hippocampus cells confirmed the cell type specific molecular signature for both cell types. qNSCs and aNSCs both express the sorting marker Prom1 and Slc1a3 (data not shown) but differentially express proliferation markers, a hallmark of aNSC and qNSC discrimination. The proliferation marker mKi67 and Notch1 were both highly expressed in aNSC but not in qNSC (Supplementary Fig. 2b) [29, 30].

A significantly greater number of aNSCs were isolated from the DG of *EGFL7*-/- mice (5.6 ± 1.65%) compared to WT litters (2.2 ± 0.53%), verifying a higher frequency of qNSCs entering the cell cycle in vivo (Fig. 2d). This suggests that either the quiescence phase of NSCs was shorter upon EGFL7 KO or proliferation of NSCs became triggered by pro-mitotic cues.

The number of proliferating cells in the hippocampus at different time points was visualized in vivo via intraperitoneal (ip) application of two different thymidine analogues: 5-iodo-2'-deoxyuridine (IdU) and 5-chloro-2′-deoxyuridine (CldU). Both markers incorporate only into the DNA of proliferating cells. IdU was injected 24 h before and CldU 1 h before subjects were sacrificed to discriminate among cells just entering S phase (CldU+/IdU-, Fig. 2e, f) or continuously proliferating after 24 h (Ki67+/IdU+; Fig. 2e, g). We did not detect any CldU+ /IdU+ cells, which means that the IdU + cells have not yet re-entered the cell cycle. We did, however, observe more CldU+/IdU- cells in the *EGFL7-/-* mice. Previous work [31] indicates proliferation length differences in neural stem and precursor cells of the hippocampus. The quickest cells had a cycling time of 22.8 ± 0.5 h, which reflects the duration of our analysis. Thus, we interpret the presence of CldU+/IdU- cells as reflecting overall more proliferating cells in *EGFL7-/-* mice. Specifically, we detected twice the number of proliferating cells in the SGZ of *EGFL7*-/- mice as compared to WT litters at both time points, supporting the conclusion that the pool of proliferating cells in the hippocampus becomes sustainably enhanced in adult mice upon EGFL7 deletion.

### EGFL7 deletion stimulates neurogenesis in the hippocampus by expansion of newborn neurons

**Fig. 3:**
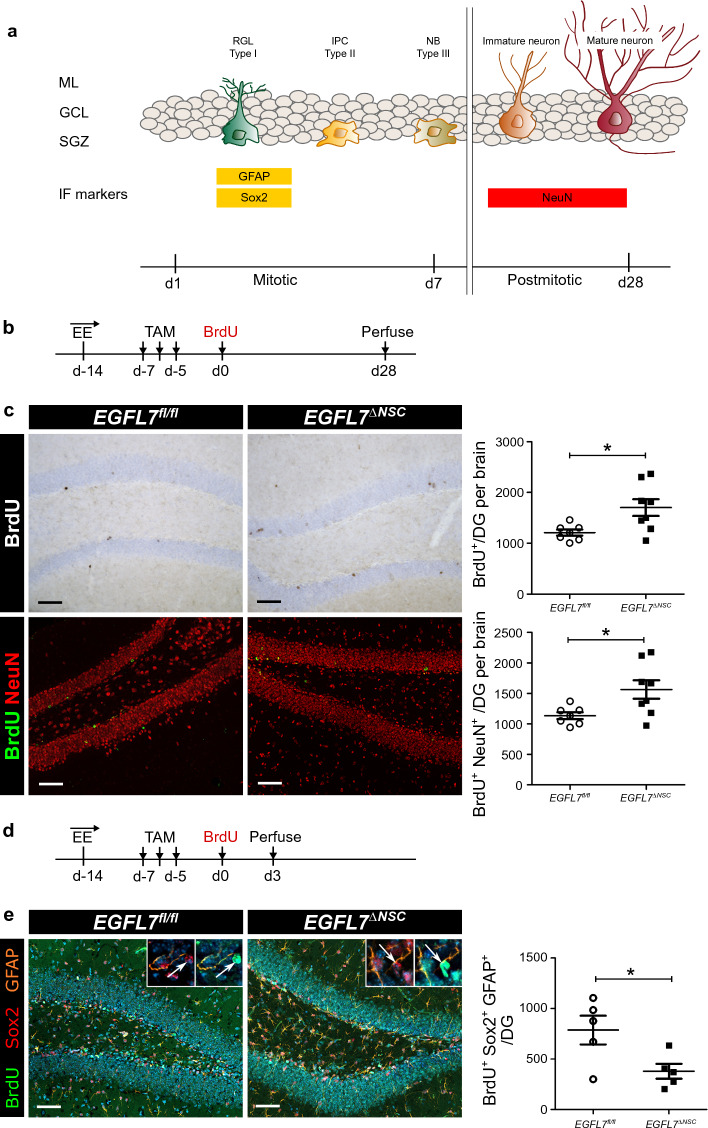
EGFL7 deletion in neural progenitors phenocopies the global EGFL7 knock-out. **a** Markers used in immunoperoxidase and immunofluorescence analyses (IF) to label specific cell types of adult neurogenesis in the dentate gyrus (DG): GFAP + Sox2 + for radial glial (RGL) cells, NeuN for mature neurons. **b** Experimental paradigm of applied neurogenesis assays (long-term course). **c** Quantification of BrdU + cells in the DG per brain were found to be increased in EGFL7ΔNSC mice (1700 ± 165; *n* = 8 versus 1207 ± 59,68 cells per DG in EGFL7 fl/fl; *n* = 7; *p* = 0.0199); Phenotyping of BrdU + cells with neuronal marker (NeuN). Quantification of newborn neurons (BrdU + /NeuN+) revealed a significant upregulation in EGFL7 del;Nestin-CreERT2 mice (EGFL7ΔNSC)C (1564 ± 152,0 per DG in EGFL7 fl/fl control; *n* = 8 versus 1135 ± 56,13; *n* = 7; *p* = 0.0263). **d** Experimental paradigm of applied neurogenesis assays (short-term course). **e** The number of BrdU + Sox2 + GFAP + cells was decreased in EGFL7ΔNSC mice (379.2 ± 73.54; *n* = 5 versus 787.2 ± 141in EGFL7 fl/fl; *n* = 5; *p* = 0.0334). Statistical analysis was performed by Mann–Whitney *U*-test. Data are represented as mean ± SEM; *p < 0.05; Scale bars represent 60 µm

To determine the influence of EGFL7 in NSCs on adult neurogenesis in the hippocampus in vivo, we quantified neural stem/progenitor cells (NSPCs) and newborn neurons in conditional *EGFL7* *fl/fl;Nestin-CreERT2* mice and *EGFL7* *fl/fl* littermates. *EGFL7* *fl/fl;Nestin-CreERT2*, a tamoxifen-inducible cell type-specific EGFL7 KO system based on the expression of *Nestin-CreERT2*, was applied allowing for the conditional deletion of EGFL7 in NSCs (radial glial-like cells, type 1 cells). *EGFL7* *fl/fl;Nestin-CreERT2 as well as EGFL7 fl/fl* mice received three injections of tamoxifen ip at 6 weeks of age. Mice were housed in cages with an enriched environment (EE) and sacrificed 3 d and 28 d after administration of BrdU to examine either early stages (represented by NSPCs) or later stages (newborn neurons) of neurogenesis (Fig. 3b,d).

We found a significant induction of BrdU + cells in the DG per brain of *EGFL7* *fl/fl;Nestin-CreERT2* as compared to *EGFL7* *fl/fl* mice (Fig. 3c). For phenotyping of BrdU + cells, double immunofluorescence was performed. The proportion of newborn neurons (BrdU + /NeuN +) was around 94% in *EGFL7* *fl/fl;Nestin-CreERT2* mice and 92% in *EGFL7* *fl/fl*. The absolute numbers of newborn neurons in the DG per brain of EGFL7 fl/fl and EGFL7 fl/fl;Nestin-CreERT2 mice are shown (Fig. 3c). The results revealed an increased proportion of neuronal differentiation in *EGFL7* *fl/fl;Nestin-CreERT2* mice. To determine whether the increase in proliferation was driven primarily by an expansion of radial glial cells exiting quiescence, we used double immunofluorescence to identify type 1 cells (BrdU + Sox2 + GFAP +) (Fig. 3e). We found a decrease in the amount of type 1 cells in *EGFL7* *fl/fl;Nestin-CreERT2* mice in comparison to *EGFL7* *fl/fl* littermates. Taken together, we observed that the number of NSPCs was increased upon EGFL7 KO in aNSCs, while the number of type 1 cells was reduced, which may indicate that this cellular development stage, characterized by rapid proliferation, was progressing at a higher rate. Overall, our findings thus demonstrate that neural stem/progenitor cells are especially sensitive to EGFL7 KO in adult hippocampal neurogenesis.

### Notch signaling in hippocampus-derived aNSCs/NPCs

EGFL7 has been identified as a non-canonical Notch ligand that affects Notch signaling in vivo and in vitro [27, 28]. Stem cells were isolated from WT and *EGFL7-/-* hippocampi and grown as neurospheres. With respect to the 2 time points, the cells were plated in clonal density and grown for either 2 or 5 days before they were harvested. RNA was extracted and transcribed to cDNA, and qRT-PCR was performed to define potential candidates responsible for differential effects of EGFL7 on NSCs and their progeny in the hippocampus, Notch signaling components were quantified by qRT-PCR in hippocampus-derived aNSCs/NPCs (Supplementary Fig. 3). Prominent levels of the Notch receptors 1–3, the neuron-specific Delta/Notch-like EGF-related receptor (DNER), the canonical ligand Jagged1, the non-canonical ligands Dlk2 and Dll3 as well as the Notch reporter genes Hes1, Hes5, Hey1 and Hey2 were detected (Supplementary Fig. 3a,b). Note: The expression levels of Notch4, Dll1, Jagged2, Dlk1 and Hes3 were smaller than 0.05 as normalized to EGFL7 and were therefore not included in the diagram. Lastly, relevant expression levels of lunatic fringe (LNFG), manic fringe (MFNG) and radical fringe (RFNG)*,* modifiers of Notch receptor fucosylation, were detected in hippocampus-derived aNSCs/NPCs. However, no significant differences in the expression levels of these Notch components were detected upon deletion of EGFL7, suggesting that EGFL7 does not significantly exert its function in hippocampus-derived aNSCs/NPCs via Notch signaling.

### The cytokine VEGF-D is upregulated in *EGFL7-/-* mice and increases neurogenesis

**Fig. 4:**
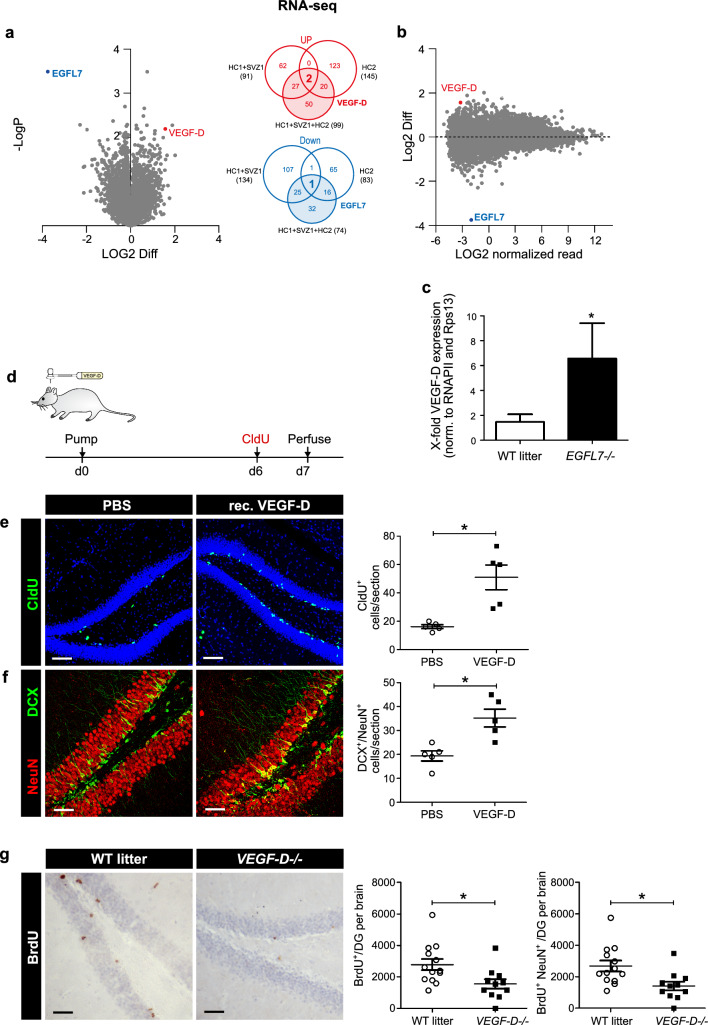
Upregulation of VEGF-D upon EGFL7 knock-out and VEGF-D neurogenic stimulation in vivo. **a** Volcano and **b** MA plots of RNA-sequencing-based transcriptomics in neural stem and precursor cells confirmed EGFL7 knock-out and identified an upregulation of the cytokine VEGF-D (*n* = 3 for HC1, SVZ1 and *n* = 4 for HC2; two datasets). Volcano plots illustrate the Log2 difference (i.e., fold change) versus -logP of the *t*-test. MA plots display the LOG2 difference versus the mean expression level (LOG2 RPKM). The VENN diagrams show the agreement of experiments 1 and 2 and the combined analysis of regulated candidates, based on P-value and fold change. **c** Upregulation of VEGF-D upon EGFL7 knock-out was confirmed by quantitative reverse transcriptase-polymerase chain reaction (6.56 ± 2.85 versus 1.47 ± 0.61 in wild-type control (WT); *n* = 3; *p* = 0.039). Statistical analysis was performed by Student’s *t*-test. **d** Recombinant, purified VEGF-D was infused in the dentate gyrus of mice using osmotic pumps; Experimental paradigm to study neurogenesis in this setup. **e** CldU staining in the dentate gyrus revealed a significant increase in proliferation upon infusion of recombinant, purified VEGF-D (51.00 ± 19.43 versus 16.20 ± 3.03 cells per section in wild-type controls (WT); *n* = 5, *p* = 0.0079). **f** Maturation of adult-born neurons was enhanced by VEGF-D (35.2 ± 8.29 versus 19.40 ± 4.72 cells per section in WT; *n* = 5; *p* = 0.0159). **g** Quantification of BrdU + cells in DG revealed a significant downregulation in *VEGF-D-/-* mice (1552 ± 299*,* *n* = 11 versus 2783 ± 354 in WT littermates*,* *n* = 13; *p* = 0.0164); Phenotyping of BrdU + cells with neuronal marker (NeuN). Quantification of newborn neurons (BrdU+/NeuN+) revealed a significant downregulation in *VEGF-D-/-* mice (1412 ± 272,4; *n* = 11 versus 2689 ± 344,8 per DG in WT littermates; n = 13; p = 0.0098). Statistical analysis was performed by Mann–Whitney *U*-test. Data are represented as mean ± SEM; *p < 0.05; Scale bars represent 60 µm

The above data suggested that EGFL7 loss expanded the NSPCs pool in the SGZ of the hippocampus, stimulating neurogenesis. The presence of EGFL7 thus appears to have a regulatory role in NSPCs proliferation. However, potential molecular influences underlying this function of EGFL7 remained unclear. Based on this, we performed RNA sequencing experiments on aNSCs/NPCs derived from *EGFL7*-/- mice and WT littermate controls (Fig. 4a, b). This particular experiment consisted of two sequential studies. Comparisons of regulated gene sets i) confirmed the deletion of EGFL7, ii) revealed a reciprocal expression of VEGF-D (Venn diagrams) and iii) identified additional top regulated candidates (Supplementary Fig. 4). Transcriptomic results on VEGF-D were confirmed by qRT-PCR, which exhibited a sixfold increase of VEGF-D in *EGFL7*-/- aNSCs/NPCs as compared to WT litters (Fig. 4c).

To assess the functional relevance of VEGF-D in the hippocampus, recombinant purified VEGF-D protein was infused via osmotic pumps into the ventricle of 8-week-old WT mice for 7 days (Fig. 4d). In parallel, proliferating cells were labeled in vivo by administration of the base analog CldU ip at day 6 (Fig. 4d). At day 7 animals were sacrificed by transcardial perfusion, brains were removed, fixed, and hippocampi sliced to characterize proliferating cells and immature neurons by immunofluorescence analyses as described above. Staining revealed a significant increase in CldU^+^ cells following the administration of recombinant VEGF-D (Fig. 4e). Additionally, the amount of adult-born immature neurons (DCX+/NeuN+) increased upon VEGF-D administration for one week (Fig. 4f). Finally, to determine the direct influence of VEGF-D on adult hippocampal neurogenesis in vivo, we utilized a VEGF-D knock-out mouse model (*VEGF-D-/-)*, quantifying BrdU + cells in the DG. The analysis of adult neurogenesis by BrdU+/NeuN+ cells in the DG revealed a pronounced reduction in adult-born neurons in *VEGF-D-/-* mice (Fig. 4g), thus demonstrating a clear downregulation of hippocampal neurogenesis as a result of VEGF-D loss. Taken together, these data support the conclusion that VEGF-D stimulates neurogenesis in the hippocampus and indicates a potential role of EGFL7 in regulating the function of this cytokine.

### EGFL7 deficiency improves learning and memory

**Fig. 5:**
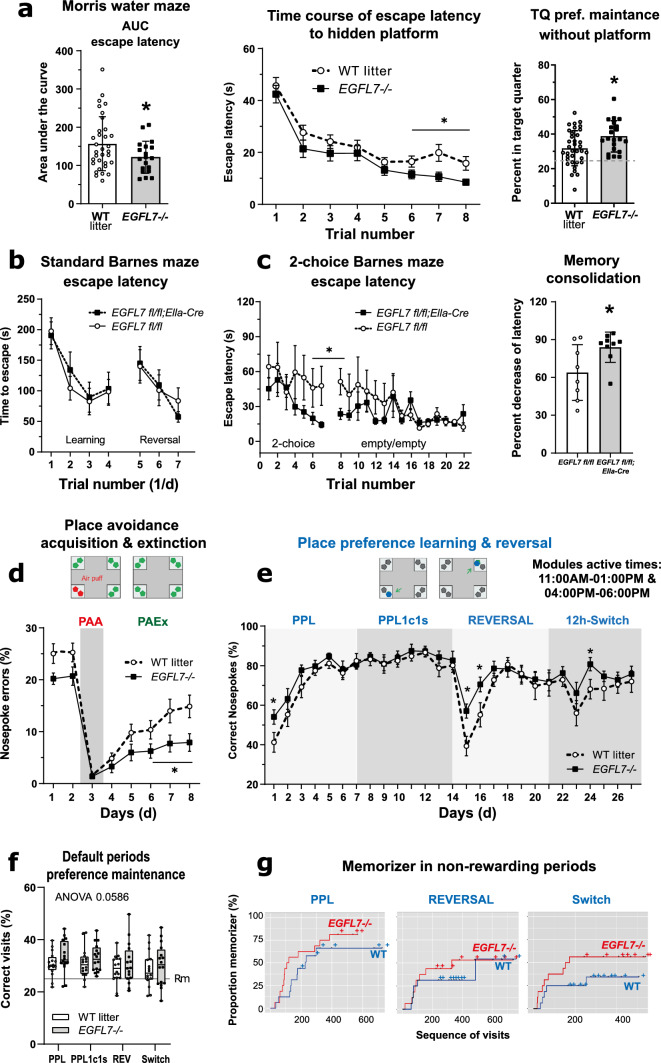
Loss-of-EGFL7 improves spatial learning and memory. **a** In the Morris water maze, *EGFL7*-/- mice were faster in finding a hidden platform. Escape latency was reduced considering the full time course (AUCs) and late trials. The proportion of time spent in the platform quarter after its removal was increased. Sample sizes were *n* = 19 for *EGFL7*-/- mice and *n* = 33 for wild-type litters (WT). **b**, **c** In the classical Barnes maze, escape latencies were similar (*n* = 12 per genotype), but in the two-choice Barnes maze test, *EGFL7 fl/fl;EIIa-Cre* mice learned faster to prefer the rewarding box and escaped faster than control mice. Sample sizes were *n* = 8 for *EGFL7 fl/fl;EIIa-Cre* mice and *n* = 9 for *EGFL7 fl/fl* control. **d** In the IntelliCage, *EGFL7*-/- mice maintained longer avoidance of a previously punished corner (air puff), as revealed by a reduced proportion of nosepoke errors (*n* = 16 per genotype). **e** During preference learning in active module times (11:00 AM – 01:00 PM and 04:00 – 06:00 PM every day), *EGFL7*-/- mice showed faster relearning to prefer a specified corner upon switching to the opposite side (reversal learning), indicated by a higher proportion of correctness of nosepokes (*n* = 16 per genotype; overall activity is shown in Supplementary Fig. 4). **f**, **g** During periods in which the learning modules were inactive (doors remain closed, "default-times"), *EGFL7*-/- mice maintained a higher preference of rewarding corners and the proportion of "memorizers" was higher, the latter defined as 35% correctness of corner visits (random = 25%). Time course data are represented as mean ± SEM, summarizing data as mean ± SD. The boxes show the interquartile range, the line is the median, and whiskers are plotted minimum to maximum. Scatters represent individual mice. Time courses were compared by two-way ANOVA and subsequent post-hoc *t*-tests to assess genotype differences at specific time points without multiplicity adjustment for between group comparisons, AUCs were compared with two-sided unpaired *t*-tests; *p < 0.05

The above findings raised the question of whether upregulated adult neurogenesis resulting from EGFL7 loss is of physiological relevance for living animals, prompting us to assess learning and memory in behaving mice. In the Morris water maze, *EGFL7*-/- mice reached the hidden platform faster than their WT littermates (Fig. 5a), which was evident by comparing the area under the curves (AUCs) and time courses. Genotype differences manifested in the later trials suggesting a stronger memory consolidation and possibly, a stronger stimulating effect of daily exercise via swimming. Indeed, *EGFL7*-/- mice retained a stronger preference for the target quarter after removal of the platform (right panel). There were no differences in finding the visible platform or swimming velocity (Supplementary Fig. 5a). Furthermore, body weight, indicating general health, was comparable among animals throughout experiments (Supplementary Fig. 5b). The Morris water maze is influenced by the swimming dislike of mice and therefore, further spatial tests were done using a Barnes maze (Fig. 5b,c). There was no difference in the classical test requiring escape through one target hole into the escape box during acquisition or during reversal (Fig. 5b), but in a two-choice award-based paradigm, *EGFL7*-/- mice were faster to escape.

Based on these results, specific tests were designed in the IntelliCage, which provides a PC-controlled observation of groups of mice without handling. The overall activity of visits, nosepokes and licks was very similar in both genotypes throughout the observation period lasting up to midlife (Supplementary Fig. 5c). There was also no difference in the acquisition of avoidance (Fig. 5d) or corner preferences (Fig. 5e). However, *EGFL7*-/- mice retained longer memory of a previously "punished" forbidden corner as indicated by lower errors (Fig. 5d), and they were faster to re-establish preference of a rewarded corner after its removal to the opposite side of the cage (Fig. 5e). This reversal learning period is the most critical and most difficult part of the assay and suggested a crucial advantage that *EGFL7*-/- mice have over WT. Further analysis of the periods outside of the learning modules—in which the doors remained closed—revealed that *EGFL7*-/- mice retained stronger corner preference without re-enforcement (Fig. 5f,g), which agrees with the maze experiments and indicates that EGFL7 loss strengthens spatial memory.

## Discussion

In this study, we increased adult hippocampal neurogenesis via selective deletion of EGFL7. At the behavioral level, we demonstrated that EGFL7 loss is associated with increased spatial learning and memory. Expanding on our previous work, these findings consolidate the role of EGFL7 in neurogenesis across both neurogenic niches of the adult brain and identify the cytokine VEGF-D as a potential novel factor involved in this process in the SGZ of the hippocampus.

Firstly, the present work illustrates that EGFL7 is located in the hippocampus of both mice and humans. In the human hippocampus, we detected EGFL7 in neurons, in particular in granular cells of the DG and in the hilus. Endothelial cells of larger blood vessels also displayed immunoreactivity as expected [24]. Expression data also substantiate that EGFL7 is expressed by hippocampal neurons in humans. Our expression studies in mice using FISH further confirmed EGFL7 in granular cells and revealed that NSCs, NPCs and neurons synthesized the majority of EGFL7 in the SGZ. Secondly, we confirmed our hypothesis that the loss of EGFL7 would result in upregulation of progenitor proliferation and differentiation. Specifically, we first showed that neurospheres, grown in vitro from the DG of *EGFL7-/-* mice grew significantly larger than WT controls, indicating increased cell proliferation. This finding was verified in vivo by flow cytometry analysis, identifying double the number of aNSCs in the DG of *EGFL7-/-* mice compared to WT litters. This increase in proliferating progenitors was confirmed by immunofluorescence analyses. Based on these results, we conclude that NPCs increasingly proliferate in the absence of EGFL7 in the DG. From a broader perspective, our findings demonstrate that, in addition to influencing neurogenesis in the SVZ, EGFL7 is also a distinct modulator of neurogenesis in the SGZ of the DG, with this protein’s absence upregulating the number of progenitors and adult-born neurons in both neurogenic niches of the brain.

At the molecular level, we previously identified EGFL7 as a neurovascular regulator of NSCs in the SVZ, governing olfactory perception and behavior. Specifically, EGFL7 antagonizes Jagged1-induced Notch signaling [27] while stimulating Dll4-induced [28] activation of Notch receptors in NSCs. However, in contrast to our assumption that the influence of EGFL7 would be consistent across both neurogenic regions, we demonstrate here that the molecular mechanism underlying this protein’s impact on neuronal differentiation and maturation in the SGZ of the hippocampus differs from that of the SVZ. Specifically, in the current work, our transcriptomic analysis of NSCs of the SGZ revealed an upregulation of VEGF-D upon loss of EGFL7. However, no significant differences in the expression levels of Notch components were detected in *EGFL7-/-* mice. This indicates that EGFL7 does not significantly exert its regulatory function in hippocampal aNSCs and NPCs via Notch signaling. Thus, despite EGFL7’s involvement in regulating neurogenesis in both neurogenic niches, our previous findings combined with the current results argue for regional specificity in the molecular mechanisms underlying this process.

To understand this, it is important to highlight that the local microenvironment significantly differs between the two neurogenic niches in the mammalian brain. In the SGZ, NSCs contact not only nearby neurons of the granular cell layer but also local interneurons and mossy cells, thus forming a substantial neural network governing local neurogenesis [32]. In contrast, NSCs in the SVZ lack robust contact to a large number of neurons and instead are located intimate to blood vessels and the lateral ventricles [33]. Neurogenesis itself further differs between these regions. Neuroblasts of the SVZ migrate approximately 5 mm in mice [34, 35] before they reach the OB, differentiate into inhibitory interneurons, and connect to the olfactory microcircuit [36]. In contrast, adult-born neurons and their progeny in the SGZ mostly stay local and connect with long axons to GCs of the CA3 region of the hippocampus. Their apical dendrites reach into the molecular layer of the hippocampus and form a characteristic dendritic tree allowing them to receive input from the entorhinal cortex via the performant path [37]. These regional differences ultimately affect the local milieu with which the NSCs and their progeny interact. Taken together, these differences in microenvironment, coupled with clear differences in cellular development, migration and functional outcomes, point towards potential significant variations in the mechanisms underlying neurogenesis between the SGZ and the SVZ.

Our transcriptomic analysis identified VEGF-D as a candidate for inducing upregulation of neurogenesis in the SGZ in the absence of EGFL7. VEGF-D is a cytokine produced by both neurons and other cell types in the adult brain [38]. NSCs in the DG express VEGF-D’s receptor VEGFR-3, a receptor tyrosine kinase activating the AKT and ERK pathways upon ligand binding and thereby controlling cell fate and proliferation [39]. To substantiate and extend our transcriptomic analysis we infused purified recombinant VEGF-D in the DG of WT mice, which phenocopied the increase in adult-born neurons seen in the *EGFL7-/-* mice, providing further evidence that VEGF-D acted downstream of EGFL7 to upregulate neurogenesis. Following this, we then utilized a *VEGF-D-/-* mouse model in whom EGFL7 was intact, demonstrating that loss of VEGF-D results in a pronounced downregulation of neurogenesis. Thus, our results indicate that VEGF-D represents a novel, molecular driver of neurogenesis in the SGZ of the hippocampus that correlates with the expression of EGFL7. In other words, here we show that both EGFL7 and VEGF-D are involved in hippocampal neurogenesis. It is, however, important to note that additional factors may mediate or moderate the relationship between these proteins. Therefore, a goal of future research will be to illuminate the causal mechanisms underlying the relationship between EGFL7 and VEGF-D with respect to neurogenesis in the hippocampus.

In addition to the molecular and cellular impacts of EGFL7 loss in the SGZ, upregulation of hippocampal neurogenesis in *EGFL7-/-* mice was also associated with effects on behavior. Previously, neurogenic upregulation in the SVZ due to EGFL7 loss resulted in pronounced deficits in olfactory perception and behavior [28]. Here, *EGFL7-/-* mice displayed subtle but distinct and reproducible advantages in a number of reward- and avoidance-based memory tasks during midlife. The predominant phenotype across behavioral studies was a stronger maintenance of memory. This manifested in i) shorter paths towards platform or shelter in late training trials in the Morris water maze and Barnes maze, ii) stronger maintenance of platform quarter preference after removal of the platform, iii) longer avoidance of air puff-punished corners and iv) stronger adherence to preferred corners in non-rewarded periods in the IntelliCage. Overall, these tests point towards a longer-lasting spatial memory and an improved memory consolidation in *EGFL7-/-* mice, parameters affected by adult neurogenesis in the hippocampus by the modulation of pattern separation [40–43]. Importantly, we note, however, that we cannot rule out the possibility that other cells (e.g., astrocytes, microglia) apart from neurons may be playing a role in behavioral readouts. In addition to advantages in reward- and avoidance-based memory, *EGFL7-/-* mice were also faster in reversal learning, a type of cognitive flexibility principally mediated by the hippocampus [44–46]. Interestingly, reversal learning represents the most critical and difficult part in learning tasks, requiring a switch of strategy and the search for alternative solutions.

Taken together, our current and previous results indicate that EGFL7 loss, which we demonstrate is associated with the upregulation of neurogenesis, produces divergent functional outcomes across the two neurogenic regions of the rodent brain. Specifically, EGFL7 loss in the SVZ produced a corresponding loss of olfactory function, while in the SGZ we observed a gain in function related to spatial learning and memory. This leads to the question of why such a functional divergence is observed. Maintenance of neurogenesis and spine plasticity in the adult hippocampus are proposed to preserve cognitive youthfulness and represent an important path towards healthy brain aging [38, 47, 48]. Furthermore, from a clinical perspective the overexpression of pro-neurogenic factors in mice has been shown to restore spine loss and cognitive deficits in certain animal models of dementia [49, 50]. However, hippocampal neurogenesis is a double-edge sword, as the development and integration of adult-born neurons remodels hippocampal circuits and, over time, this integration can degrade established memories (i.e., forgetting) [51]. Put another way, there is a trade-off between *plasticity* and *stability* in the networks of the hippocampus and rampant proliferation may harm existing memory structures, despite fostering new memories and learning, as we observed in this study. Indeed, we see a similar outcome from increased neurogenesis in the SVZ due to EGFL7 loss, with an overabundance of adult-born inhibitory interneurons (important for olfactory pattern discrimination) disrupting olfactory bulb networks leading to behavioural deficits such as mice not reacting to fox urine extract [28]. It follows that regulatory mechanisms are thus required to control the rate of adult-born neurons entering into these networks. Our results strongly suggest that EGFL7 is such a regulator of neurogenesis, imposing a cap on unfettered neurogenic proliferation in both neurogenic niches. Moreover, given local variation in the microenvironments of the SVZ and SGZ, and the corresponding differences in the required network regulation and function, our findings indicate that EGFL7 potentially exerts its regulatory effects via different molecular mechanisms (i.e., Notch signaling vs. VEGF-D) across these neurogenic regions.

Finally, it is important to consider that this double-edged aspect of upregulating neurogenesis is for healthy brains and the potential to (artificially) increase the number of adult-born neurons in disease states (in which network stability is already disrupted) may still offer novel therapeutic avenues for improving brain function. Ultimately, EGFL7 is an unusual and rare case of a single gene that, when removed, upregulates neurogenesis and produces measurable improvements in certain hippocampal functions. Immediately, this raises the question of the role of EGFL7 in the human brain, as our results indicate that EGFL7 is present not only in the granular cells of mice but also in neurons of the human hippocampus. Moreover, our findings suggest that VEGF-D may be acting as a downstream facilitator of EGFL7 to upregulate neurogenesis and could thus represent a clinical target in humans for improving hippocampal function in disease states. Indeed, VEGF-D has been described as supporting dendritic and synaptic plasticity as evoked by exercise [40], as well as preserving the dendritic architecture and facilitating functional recovery in a murine stroke model [38]. Overall, we extend previous findings by demonstrating that EGFL7 and VEGF-D are novel modulators of neurogenesis, with ablation of EGFL7 upregulating—and ablation of VEGF-D downregulating—the number of adult-born neurons in the SGZ of the hippocampus. However, it is also important to note that previous finding have revealed cell type- and species-specific transcriptomic properties influencing neurogenesis in the hippocampus, including differences between mice and humans [52]. Moreover, our normalized comparison of EGFL7 expression in mice and humans demonstrates an approximately two-fold greater expression of this protein in humans compared to mice. It is possible that these species level differences in expression may produce differences in the influence of EGFL7 on hippocampal neurogenesis. Thus, a goal of future work will be to i) reveal the underlying mechanisms by which EGFL7 may be influencing VEGF-D expression in the hippocampus and ii) investigate whether the targeted downregulation of EGFL7 or application of VEGF-D in humans may be used to upregulate SGZ neurogenesis and delay or mitigate the cognitive decline resulting from the impact of neurodegenerative diseases of the hippocampus.

## Materials and methods

### Animals

Animal experiments were approved by the Landesuntersuchungsamt Koblenz, Rhineland‐Palatinate, Germany, the Regierungspräsidium Darmstadt, Hesse, Germany and the Landesdirektion Chemnitz, Saxony, Germany (permit number TVA G16-1–002 and TVV 56/2020). Experiments were conducted according to the German Animal Welfare Law and the Directive 2010/63/EU for the protection of animals used for scientific purposes and followed the ARRIVE and GV-SOLAS guidelines for research in behaving animals. Mice were housed in climate-controlled, pathogen‐free conditions at the Transgenic Animal Research Center (TARC, University Medical Center Mainz, Germany), the central animal research facility (ZFE, University Hospital, Frankfurt, Germany) and the Experimental Center of the Medical Theoretical Center (MTZ, Medical Faculty Carl Gustav Carus, Dresden, Germany) on a 12 h light–dark cycle with free access to food and water.

### Human tissue

We received blank sections from the Tumor and Normal Tissue Bank of the UCC Dresden for the analysis of the human hippocampus (project number: 2020_34).

### Mouse lines

C57BL/6 J inbreed mice (control WT mice) were purchased from Janvier Labs (Le Genest-Saint-Isle, France).

Constitutive *EGFL7-/-* mice were generated and kindly provided by Weilan Ye, (Genentech, San Francisco, USA) and applied as previously described [53]. KO was achieved by insertion of a retroviral gene trap vector upstream of intron 2 of the *Egfl7* gene. The insertion led to silencing of EGFL7 translation as transcripts initiated from the inserted vector contain stop codons in all three frames.

*EGFL7 fl/fl;EIIa-Cre*. Conditional *EGFL7* *fl/fl* mice were generated by insertion of loxP cassettes upstream of exon 3 and downstream of exon 7 (ingenious targeting laboratory, Ronkonkoma, USA). This way Cre-mediated recombination removed all putative start codons from the *Egfl7* gene [28]. *EGFL7* *fl/fl* animals were crossed with EIIa-Cre pan-deleter animals [54] expressing Cre under the adenovirus promoter EIIa, allowing for the deletion of EGFL7 in, among others, germ line cells. This alternative constitutive EGFL7 KO model was applied in a set of behavioral studies to exclude strain-specific artifacts.

*EGFL7 fl/fl;Nestin-CreERT2*. Conditional *EGFL7* *fl/fl* mice [28] were crossed with *Nestin-CreERT2* animals [55], which express a CreERT2 fusion protein under the control of the nestin (*Nes*) promoter for tamoxifen-induced deletion of EGFL7 in neural stem cells and early precursor cells (NPCs). *Nestin-CreERT2* mice were provided by Beat Lutz (Institute of Physiological Chemistry, Mainz, Germany) and originated from Günther Schütz’s lab (DKFZ, Heidelberg, Germany) [55].

*VEGF-D-/-* mice were provided by Kari Alitalo (Translational Cancer Biology, Helsinki, Finland) and originated from Marc Achens’ lab (Ludwig Institute for Cancer Research, Melbourne, Australia). They were created by insertion of a lacZ-pgk-neo cassette to replace the signal sequence in exon 1 downstream of the translation start site leading to deletion of vegfd gene located on the X chromosome as described previously [56].

### Mouse treatments

BrdU, CldU: A 10 mg/ml solution of BrdU or CldU was prepared and 100 µl of injection solution were injected ip. IdU: 500 µl of a 2 mg/ml solution were injected IP. Hence, mice received about 1 mg base analog per animal (approximately 40 mg/kg). Hence, mice received about 1 mg base analog per animal (approximately 40 mg/kg). Tamoxifen: Mice received daily injections of tamoxifen in peanut oil/ethanol (9:1) as a vehicle ip at a dose of 3 mg/20 g body weight for three consecutive days.

### Osmotic pump implantation and intraventricular infusion

8-week-old male C57BL/6 J mice (*n* = 6) were anesthetized by the administration of a ketamine–xylazine mixture ip (100 μl per 10 g body weight, ketamine 200 mg/kg, xylazine 20 mg/kg, both in a 0.9% sodium chloride solution). At the surgical tolerance stage, an incision was made with a scalpel above the position of the bregma, and the skull was exposed. A 1 mm diameter hole was drilled at the following coordinates: 0 mm anteroposterior, 0.8 mm lateral to bregma. Subsequently, a catheter was placed through the hole 2 mm below the surface of the skull into the lateral ventricle and connected to a mini-osmotic pump (2001, Alzet, Cupertino, CA, USA) which was placed into a subcutaneous skin dorsal fold. The pump delivered 1 µg/d of recombinant purified VEGF-D (R&D systems, Minneapolis, MN, USA) into the ventricle for 7 d at 1 µl/h. The wound was closed and mice were allowed to recover. Mice received IdU solution (200 mg/kg body weight) ip at d1 or CldU solution (200 mg/kg body weight) at d6 after surgery. Mice were sacrificed and tissue prepared at d7.

### Tissue preparation

Mice were anesthetized by the administration of a ketamine–xylazine mixture ip (100 μl per 10 g body weight, ketamine 450 mg/kg, xylazine 48 mg/kg, both in a 0.9% sodium chloride solution). Subsequently, mice were perfused transcardially with phosphate-buffered saline (PBS), followed by 4% paraformaldehyde in 1 × PBS. The high dose of ketamine–xylazine represents an overdose to kill the animals by perfusion.

Brains were removed, fixed in 4% paraformaldehyde in PBS at 4 °C for 24 h, subsequently transferred into 15% sucrose and finally, into 30% sucrose solution in PBS for cryoprotection. Brains were cut on a vibratome (HM650V, Thermo Fisher Scientific, Waltham, USA) to obtain 40 µm thick sections which were stored in cryoprotective solution at − 20 °C. Alternatively, brains were embedded in Tissue-Tek O.C.T. solution (Sakura Fintek, Torrance, CA, USA), frozen on dry-ice and stored at − 80 °C until further processing. Coronal cryosections were prepared at a thickness of 10 μm and stored at − 20 °C.

### Flow cytometry

Cell populations were identified by flow cytometry as previously described [57]. Animals were sacrificed by cervical dislocation; brains were immediately removed and the hippocampus was micro-dissected. Pooled tissue samples (5 mice per sample) were digested with papain plus DNase and mechanically dissociated as previously described [57].

Cells were stained for 30 min at 4 °C in the dark in PBS using the following antibodies and conjugates: qNSCs and aNSCs were discriminated by a combination of PE- anti-mGLAST (1:50, clone ACSA-1, Miltenyi Biotec, Bergisch Gladbach, Germany), APC- anti-mCD133 (1:75, clone 13A4, Invitrogen, Darmstadt, Germany), EGF, biotinylated, complexed to Alexa Fluor 488 streptavidin (Alexa Fluor™ 488 EGF complex) (1:100, Invitrogen), APC-Cy7 rat anti-mCD45 (1:200, Becton Dickinson, Franklin Lakes, NJ, USA) and APC-Cy7 anti-mTer119 (1:100, Biolegend, San Diego, CA, USA). To sort neural stem and precursor cells, neuroblasts, neurons or endothelial cells from the hippocampus by flow cytometry a fluorescence-activated cell sorting device type Aria II (BD Biosciences) was used, Alexa Fluor 488-conjugated GFAP (rabbit anti-mGFAP 1:100, clone G-A-5, Sigma), PE-Cy7-conjugated rat anti-mCD133 (1:100, clone 315-2C11, Biolegend), Biotin-xx-Conjugated EGF (1:200, Thermo Fisher Scientific) plus Streptavidin-PE (1:100, BD Biosciences, San Jose, CA, USA), eFluor405-conjugated rat anti-mCD24 (1:100, BD Biosciences), PE-conjugated rat anti-mCD31 (1:50, clone MEC133, BD Biosciences) or Thy1-GFP mice [58] were applied and cells were collected in PBS. To collect neurons from hippocampus fluorescence activated nuclei sorting was applied as described by Jaeger and colleagues [59]. Nuclei were stained with Hoechst and AF488-anti-mNeuN (1:1000, clone A60, Sigma–Aldrich) and NeuN positive nuclei were sorted in PBS.

### RNA extraction and quantitative real-time PCR

Mouse brains were mechanically disrupted using a mortar and pestle precooled in liquid nitrogen. Disruption of neurosphere cultures was achieved using Trizol Reagent (Invitrogen) according to the manufacturer's instructions. Disrupted tissues and cells were further homogenized by QIAshredder columns (Qiagen, Hilden, Germany). Cells sorted by flow cytometry were lysed in the RLT buffer (Qiagen). RNA was purified using the RNeasy mini kit (Qiagen) according to the manufacturer’s instructions. The concentration and purity of RNA were determined using the Bioanalyzer 2000 automated electrophoresis system (Agilent, Santa Clara, CA, USA). cDNA was synthesized using oligo-d(T) primers and avian reverse transcriptase (iScript cDNA Synthesis Kit, Bio-Rad, Hercules, CA, USA) according to the manufacturer’s instructions. qRT-PCR was performed using SYBR Green fluorescein mix (Thermo Fisher Scientific), using 2 ng of template cDNA and 1 pmol of gene-specific primers per reaction. PCR reaction and amplicon detection were performed by the iCycler real-time PCR system (CFX Connect RT-PCR System, Bio-Rad). Quantification of expression was normalized according to the relative levels of cDNA of the house-keeping genes RNA polymerase II (RNAPII) and ribosomal protein S13 (Rps13). Primer sequences are listed in *SI Appendix*, Table 1. Statistical analysis was performed using Student’s *t*-test.

### EGFL7 gene expression in humans and mice

RNA expression for mice (male) NeuN + FACS sorted hippocampal nuclei (*n* = 3) was measured using bulk RNA-SEQ. Raw reads were trimmed, low quality reads excluded and A/G-tails removed (Fastp). Remaining reads were then mapped against the GRCm39 (release 105) reference genome (STAR) and raw counts for each feature summarized in a count matrix. Raw fastq files for human (male) NeuN + FACS sorted hippocampal nuclei generated by bulk RNA-SEQ were downloaded from the Sequence Read Archive [60] (SRR21161826, SRR21161844, and SRR21161924). All three samples were classified as neurotypical controls. Raw reads were trimmed, low quality reads excluded and A/G-tails removed (Fastp). Remaining reads were then mapped against the GRCh38 (release 108) reference genome (STAR) and raw counts for each feature summarized in a count matrix. Genes which are present in both species were extracted and both count matrices combined. Differences in library size and composition are corrected using DESeq2’s median of ratios methods. Normalized read counts for EGFL7 are shown in Supplementary Fig. 1.

### FISH

Brain sections were cut on a cryostat at 10 µm thickness (Cryostat CM 1900; Leica), dried for 30 min at 37 °C and stored at − 20 °C until further processing. FISH was performed using QuantiGene ViewRNA ISH Cell Assay (Affymetrix, Santa Clara, CA, USA). For multiplexing, Type-1 and Type-6 probes were combined. Images were captured using a confocal microscope (SP8, Leica).

### IF and immunoperoxidase staining

Age-matched brains from male *EGFL7* *fl/fl;Nestin-CreERT2* mice and their wild-type littermates were dissected, fixed in 4% paraformaldehyde in PBS at 4 °C for 24 h, subsequently transferred into 15% sucrose and finally, into 30% sucrose solution in PBS for cut into 40 μm thick serial coronal sections on freezing microtome (HM430, Thermo Scientific). Sections were stored at − 20◦C in cryoprotectant solution (25% ethylene glycol, 25% glycerol in 0.1 M phosphate buffer, pH 7.4). Further details are provided in *SI Appendix, Materials and Methods*.

### Statistical analysis of anatomical and biological data

Data are presented as mean ± standard deviation (SD) unless otherwise stated. Statistical analyses were performed using GraphPad Prism 8.0 software (Statcon, Witzenhausen, Germany). Student’s t-test or Mann–Whitney *U-*test were used for statistical analysis of three or more biological replicates per experiment; *p* < 0.05 was considered significant. All experiments and analyses were carried out without knowledge of the genotype or treatment group. Parametric data were analyzed using an unpaired two-tailed Student’s t-test. Non-parametric data or low sample sizes were analyzed with the Mann–Whitney *U-*test. Analysis of behavioral data is explained below. Statistical methods are described in figure legends or specific experimental procedures.

### Behavioral analyses

Motor coordination and learning were tested in adult male mice as described in *SI Appendix*.

## Supplementary Information

Below is the link to the electronic supplementary material.

Supplementary file1 (DOCX 49 KB)

Supplementary file2 (PDF 66 KB)

Supplementary file3 (PDF 438 KB)

Supplementary file4 (PDF 58 KB)

Supplementary file5 (PDF 206 KB)

Supplementary file6 (PDF 143 KB)

## Data Availability

Data that support the findings of this study are available from the corresponding author upon request.

## References

[1] Seki T, Arai Y (1999). Temporal and spacial relationships between PSA-NCAM-expressing, newly generated granule cells, and radial glia-like cells in the adult dentate gyrus. J Comp Neurol.

[2] Barnea A, Nottebohm F (1994). Seasonal recruitment of hippocampal neurons in adult free-ranging black-capped chickadees. Proc Natl Acad Sci U S A.

[3] Kuhn HG, Dickinson-Anson H, Gage FH (1996). Neurogenesis in the dentate gyrus of the adult rat: age-related decrease of neuronal progenitor proliferation. J Neurosci.

[4] Kempermann G, Kuhn HG, Gage FH (1997). More hippocampal neurons in adult mice living in an enriched environment. Nature.

[5] Eriksson PS (1998). Neurogenesis in the adult human hippocampus. Nat Med.

[6] Roy NS (2000). In vitro neurogenesis by progenitor cells isolated from the adult human hippocampus. Nat Med.

[7] Knoth R (2010). Murine features of neurogenesis in the human hippocampus across the lifespan from 0 to 100 years. PLoS One.

[8] Spalding KL (2013). Dynamics of hippocampal neurogenesis in adult humans. Cell.

[9] Boldrini M (2018). Human hippocampal neurogenesis persists throughout aging. Cell Stem Cell.

[10] Ming GL, Song H (2011). Adult neurogenesis in the mammalian brain: significant answers and significant questions. Neuron.

[11] Kang E, Wen Z, Song H, Christian KM, Ming GL (2016). Adult neurogenesis and psychiatric disorders. Cold Spring Harb Perspect Biol.

[12] Anacker C, Hen R (2017). Adult hippocampal neurogenesis and cognitive flexibility - linking memory and mood. Nat Rev Neurosci.

[13] Kempermann G (2018). Human adult neurogenesis: evidence and remaining questions. Cell Stem Cell.

[14] Berdugo-Vega G (2020). Increasing neurogenesis refines hippocampal activity rejuvenating navigational learning strategies and contextual memory throughout life. Nat Commun.

[15] Kempermann G, Jessberger S, Steiner B, Kronenberg G (2004). Milestones of neuronal development in the adult hippocampus. Trends Neurosci.

[16] Lima SMA, Gomes-Leal W (2019). Neurogenesis in the hippocampus of adult humans: controversy "fixed" at last. Neural Regen Res.

[17] Yoon K, Gaiano N (2005). Notch signaling in the mammalian central nervous system: insights from mouse mutants. Nat Neurosci.

[18] Zhang R, Engler A, Taylor V (2018). Notch: an interactive player in neurogenesis and disease. Cell Tissue Res.

[19] Stump G (2002). Notch1 and its ligands Delta-like and Jagged are expressed and active in distinct cell populations in the postnatal mouse brain. Mech Dev.

[20] Ables JL, Breunig JJ, Eisch AJ, Rakic P (2011). Not(ch) just development: Notch signalling in the adult brain. Nat Rev Neurosci.

[21] Ehm O (2010). RBPJkappa-dependent signaling is essential for long-term maintenance of neural stem cells in the adult hippocampus. J Neurosci.

[22] Kopan R, Ilagan MX (2009). The canonical Notch signaling pathway: unfolding the activation mechanism. Cell.

[23] Bicker F, Schmidt MHH (2010). EGFL7: a new player in homeostasis of the nervous system. Cell Cycle.

[24] Larochelle C (2018). EGFL7 reduces CNS inflammation in mouse. Nat Commun.

[25] Nikolic I (2013). EGFL7 ligates alphavbeta3 integrin to enhance vessel formation. Blood.

[26] Jolivel V (2015). Perivascular microglia promote blood vessel disintegration in the ischemic penumbra. Acta Neuropathol.

[27] Schmidt MHH (2009). Epidermal growth factor-like domain 7 (EGFL7) modulates Notch signalling and affects neural stem cell renewal. Nat Cell Biol.

[28] Bicker F (2017). Neurovascular EGFL7 regulates adult neurogenesis in the subventricular zone and thereby affects olfactory perception. Nat Commun.

[29] Artegiani B (2017). A single-cell RNA sequencing study reveals cellular and molecular dynamics of the hippocampal neurogenic niche. Cell Rep.

[30] Shin J (2015). Single-cell RNA-Seq with waterfall reveals molecular cascades underlying adult neurogenesis. Cell Stem Cell.

[31] Brandt MD, Hubner M, Storch A (2012). Brief report: adult hippocampal precursor cells shorten S-phase and total cell cycle length during neuronal differentiation. Stem Cells.

[32] Vicidomini C, Guo N, Sahay A (2020). Communication, cross talk, and signal integration in the adult hippocampal neurogenic niche. Neuron.

[33] Li Y, Guo W (2021). Neural stem cell niche and adult neurogenesis. Neuroscientist.

[34] Lois C, Garcia-Verdugo JM, Alvarez-Buylla A (1996). Chain migration of neuronal precursors. Science.

[35] Peretto P, Merighi A, Fasolo A, Bonfanti L (1997). Glial tubes in the rostral migratory stream of the adult rat. Brain Res Bull.

[36] Lledo PM, Merkle FT, Alvarez-Buylla A (2008). Origin and function of olfactory bulb interneuron diversity. Trends Neurosci.

[37] Toda T, Parylak SL, Linker SB, Gage FH (2019). The role of adult hippocampal neurogenesis in brain health and disease. Mol Psychiatry.

[38] Mauceri D (2020). Nasally delivered VEGFD mimetics mitigate stroke-induced dendrite loss and brain damage. Proc Natl Acad Sci U S A.

[39] Han J (2015). Vascular endothelial growth factor receptor 3 controls neural stem cell activation in mice and humans. Cell Rep.

[40] Xie Q (2019). Treadmill exercise ameliorates focal cerebral ischemia/reperfusion-induced neurological deficit by promoting dendritic modification and synaptic plasticity via upregulating caveolin-1/VEGF signaling pathways. Exp Neurol.

[41] Han W (2017). VEGF regulates hippocampal neurogenesis and reverses cognitive deficits in immature rats after status epilepticus through the VEGF R2 signaling pathway. Epilepsy Behav.

[42] Pombero A, Garcia-Lopez R, Estirado A, Martinez S (2018). Vascular pattern of the dentate gyrus is regulated by neural progenitors. Brain Struct Funct.

[43] Lin R (2019). Systemic factors trigger vasculature cells to drive notch signaling and neurogenesis in neural stem cells in the adult brain. Stem Cells.

[44] Jessberger S (2009). Dentate gyrus-specific knockdown of adult neurogenesis impairs spatial and object recognition memory in adult rats. Learn Mem.

[45] Kalm M, Karlsson N, Nilsson MK, Blomgren K (2013). Loss of hippocampal neurogenesis, increased novelty-induced activity, decreased home cage activity, and impaired reversal learning one year after irradiation of the young mouse brain. Exp Neurol.

[46] Garthe A, Roeder I, Kempermann G (2016). Mice in an enriched environment learn more flexibly because of adult hippocampal neurogenesis. Hippocampus.

[47] Kempermann G (2019). Environmental enrichment, new neurons and the neurobiology of individuality. Nat Rev Neurosci.

[48] Lepousez G, Nissant A, Lledo PM (2015). Adult neurogenesis and the future of the rejuvenating brain circuits. Neuron.

[49] Mirochnic S, Wolf S, Staufenbiel M, Kempermann G (2009). Age effects on the regulation of adult hippocampal neurogenesis by physical activity and environmental enrichment in the APP23 mouse model of Alzheimer disease. Hippocampus.

[50] Jain S (2012). Arf4 determines dentate gyrus-mediated pattern separation by regulating dendritic spine development. PLoS One.

[51] Akers KG (2014). Hippocampal neurogenesis regulates forgetting during adulthood and infancy. Science.

[52] Franjic D (2022). Transcriptomic taxonomy and neurogenic trajectories of adult human, macaque, and pig hippocampal and entorhinal cells. Neuron.

[53] Schmidt M (2007). EGFL7 regulates the collective migration of endothelial cells by restricting their spatial distribution. Development.

[54] Lakso M (1996). Efficient in vivo manipulation of mouse genomic sequences at the zygote stage. Proc Natl Acad Sci U S A.

[55] Corsini NS (2009). The death receptor CD95 activates adult neural stem cells for working memory formation and brain repair. Cell Stem Cell.

[56] Baldwin ME (2005). Vascular endothelial growth factor D is dispensable for development of the lymphatic system. Mol Cell Biol.

[57] Codega P (2014). Prospective identification and purification of quiescent adult neural stem cells from their in vivo niche. Neuron.

[58] Feng G (2000). Imaging neuronal subsets in transgenic mice expressing multiple spectral variants of GFP. Neuron.

[59] Jaeger BN (2020). Miniaturization of smart-seq2 for single-cell and single-nucleus RNA sequencing. STAR Protoc.

[60] Leinonen R, Sugawara H, Shumway M, International Nucleotide Sequence Database C (2011). The sequence read archive. Nucleic Acids Res.

