# Supplementary material for: EGFL7 loss correlates with increased VEGF-D expression, upregulating hippocampal adult neurogenesis and improving spatial learning and memory
Source: Cell Mol Life Sci. 2023 Jan 30;80(2):54. doi: 10.1007/s00018-023-04685-z (PMC9886625; doi:10.1007/s00018-023-04685-z)
Supplement: Supplementary file 1 — Supplementary file1 (DOCX 49 KB) [file 18_2023_4685_MOESM1_ESM.docx]

**Supporting Information**

**SI Materials and Methods**

## Neural stem cell assays

Primary neurosphere cultures were prepared from adult mice (8-12 weeks old) as previously described (1) Mice were sacrificed by cerebral dislocation. Brains were removed and the forebrain was opened under sterile conditions to remove the hippocampus. Tissue was enzymatically dissociated by incubation in Leibowitz L-15 medium containing 0.8 mg/ml papain and 0.5 mM Ethylenediaminetetra­acetic acid (EDTA; dissociation medium, pre-activated at 37°C for 30 min) at 37°C for 30 min. After centrifugation for 3 min at 300 *g*, the supernatant was removed and the tissue was resuspended in ovomucoid inhibitor (Worthington, Lakewood, CO, USA). Cells were triturated with a fire-polished Pasteur pipette, collected by centrifugation for 3 min at 300 g, resuspended in NSC medium (DMEM/F-12 medium containing 1 mM HEPES, 10 μg/ml penicillin, 10 μg/ml streptomycin, 1 x B27 supplement, 20 ng/ml EGF and 20 ng/ml FGF) and were plated in T75 flasks. Cells were incubated at 37°C and 5% CO_2_ and were passaged every 5 d by enzymatic dissociation with accutase (PAA, Egelsbach, Germany) at 37°C for 10 min under vigorous shaking. For analysis of sphere size and number, neurospheres were grown for 10 d *in vitro* at clonal density. The number of formed spheres and their diameters were determined using an IX70 microscope (Olympus, Hamburg, Germany). Statistically significant differences between genotypes were calculated using two-sided, unpaired Student’s t-tests.

## Cell cycle analysis

Cell cycle analysis of ploidy by flow cytometry was performed using Hoechst 33342 dye (Sigma Aldrich, St. Louis, MO, USA). HC-derived NSCs were incubated in 10 μg/ml Hoechst 33342 solution for 10 min at 37°C in culture medium. Incorporated Hoechst 33342 was excited using an UV Laser. Cell scatters were gated according to fluorescence intensity and plotted as histograms. The area parameter histogram was used to determine the percentage of cells in G1, S or G2/M phase. Mann-Whitney *U*-test was used for statistical analysis.

## IF and immunoperoxidase staining

For BrdU detection every sixth brain section from *EGFL7 fl/fl;Nestin-CreERT2* at 10 weeks of age was used. For BrdU detection, 40 µm free floating sections were washed and quenched with 0.6% H_2_O_2_ for 30 min, incubated in 2.5 N HCl for 30 min at 37°C followed by 1 h blocking with TBS++ (10% rabbit serum, 0.2% Triton-X 100 in TBS) and incubation in monoclonal rat anti-BrdU antibody (1:1,000; abcam 6326) overnight at 4°C in TBS+ (3% rabbit serum, 0.2% Triton-X 100 in TBS). To determine the total number of labeled cells, the peroxidase method was used with biotinylated rabbit anti-rat antibody. Staining with rat ant-Ki67 antibody (1:500, Invitrogen, 14-5698-82) involves the same procedure without incubation with HCl.

A conventional immunohistochemical procedure was followed using a commercially available detection system (Vectorstain Elite Kit, Vector Laboratories, Burlingame, USA). Peroxidase activity was visualized using 3’-3’diaminobenzidine, the sections were mounted on Superfrost slides, drying for 30 min and then tissue was counterstained by hemalum, dehydrated and cover slipped with DEPEX.

For phenotyping of BrdU+ cells, immunofluorescence was performed. Free-floating sections were washed, incubated with 2.5 N HCI for 10 min at 37°C , blocked for 1 h in 5% BSA and 0,1% Triton and incubated over night at 4◦C with the following primary antibody: sheep anti-BrdU (1:400, Biozol GTX21893). The next day, sections were washed and incubated with secondary antibody for 2 h at RT (Alexa Fluor 488 anti-sheep, 1:1,000, Invitrogen)

The next steps are washing, incubating over night at 4°C with the next primary antibody: rabbit anti-NeuN/Fox3 (1:500, abcam 104225). Day three, sections were washed and incubated with secondary antibody for 2 h at RT (Alexa Fluor 647 anti-rabbit, 1:1,000, Invitrogen).

After a final washing step, sections were incubated for 10 min in DAPI (1:5,000 in PBS; 1306, Invitrogen) and then mounted on glass slides with Fluoromount-G.

**Image Quantification**

BrdU cell quantification were performed on coded slides using widefield light microscopy with Olympus CX23. The number of immunopositive (+) cells present in the SGZ of the DG was quantified by manually counting all DAB-positive cells present within 40 μm of the granular cell layer of the DG formation (i.e., from Bregma −1.06 to Bregma −3.88).

Fluorescence images for BrdU phenotyping were obtained on a fluorescence microscope with 2D structured illumination (ApoTome.2, Zeiss). Images were processed with Zen software (Zeiss), Fiji and Illustrator CS5 (Adobe). Only general contrast and color level adjustments were made; otherwise images were not digitally manipulated. Net neurogenesis was calculated for each animal separately by generating a percentage from the proportion of BrdU+/NeuN+ cells per 100 cells counted obtained through fluorescent double labeling (BrdU+NeuN+) scaled to the total number of BrdU+ cells obtained through DAB.

## RNA-sequencing

Transcriptomic analyses consisted of two sequential experiments. The first set comprised NSCs of SVZ1 and HC1 of *EGFL7*-/- and WT mice (n=3), the second comprised neurospheres of NSCs of HC2 (n=4), only. Alignments and analyses were done for each data set and subsequently combined. RNA from NSCs was isolated after 3 d (SVZ) or 5 d (HC). RNA concentration and quality were determined using Bioanalyzer 2000 and Nanodrop. RNA-sequencing (RNA-Seq) was performed by CeGaT (Tübingen, Germany) according to standard protocols. Briefly, TruSeq RNA Sample Prep Kit (Illumina, San Diego, CA, USA) was used with 0.5 µg of total RNA for the construction of sequencing libraries. Libraries were prepared according to Illumina's instructions and SureSelectXT Mouse All Exon enrichment kits (Agilent) were used. Sequencing of 2x 100 bp was done on NovaSeq6000 (Illumina) with a sequencing depth of 50 Mio reads per sample. Starting with demultiplexed fastq.gz files, the fastqc program FASTQC (Version 0.11.5-cegat) (2) was applied to assess sample quality, and subsequently the alignment was performed with SeqMan NGen 15 (Lasergene, Madison, WI, USA) using the reference genome mm10 provided from UCSC (GRCm38) (3) as template, a minimum read length of 35 bp and automatic adapter trimming. Results were displayed with ArrayStar 15 (Lasergene) including the amount of mapped reads, target length, source length and position, strand, genes and gene IDs, annotated according to the mm10 assembly, and reads were normalized according to RPKM. Alternative DEseq2 normalization yielded equivalent results. Normalized reads were analyzed with ArrayStar, which uses general linear models to assess differential expression. Genes were filtered for at least 10 valid values out of a total of 20 samples with normalized reads > 0.05 (combined dataset) to exclude low expression genes. Data were log2 transformed, single missing values were set to an auto-calculated minimum, and results were displayed as scatter plots, MA-plots and Volcano plots, the latter showing the log2 difference, i.e., fold change (positive for upregulated genes and negative for downregulated genes) versus the -log10 of the t-test P value. The P value was set at 0.05 and adjusted according to Benjamini-Hochberg. Hierarchical clustering was employed to assess gene expression patterns using Euclidean distance metrics. Results were displayed as heat maps with dendrograms.

The differential expression analyses were first done for each experiment separately, and the agreement was assessed with Venn diagrams of top regulated genes (criteria P < 0.05, 1.5-fold change). The datasets were then combined, re-normalized and reanalyzed to gain statistical power. Functional analyses are based on the combined analysis. Gene set enrichment analyses (GSEA) ([http://www.gsea-msigdb.org](http://www.gsea-msigdb.org/)) (4) were used to assess functional implications of up- or downregulated gene networks and for gene ranking based on fold difference, P value and abundance. Leading edge 50 up- and downregulated genes according to GSEA are displayed as heatmaps.

**RNA sequencing & Data analysis of sorted cells from HC**

For all FACS sorted samples (qNSC, aNSCs and neuron nuclei with n=3 replicates) the RNA was extracted (**RNeasy Micro Kit, Qiagen**) and prepared for sequencing (SMART-Seq Stranded Kit, Takara). Fragments were then sequenced paired-end on a NovaSeq 6000 sequencer (2 x 100 bp).

Raw fastq files were trimmed, low quality reads excluded and A/G-tails removed (Fastp). Remaining reads were then mapped against the GRCm39 (release 105) reference genome (STAR) (5) and raw counts for each feature extracted. To correct between samples for differences in library size and composition, raw counts were normalized according to DESeq2’s median of ratios methods (6). Normalized read counts for target genes are visualized in a row scaled heatmap (**Supplementary Fig. 2**).

## Behavioral analyses

### Morris water maze

Spatial learning and memory were assessed in a Morris water maze (7) in a cohort of 19 *EGFL7*-/- and 33 WT litters. The bath tank (diameter 1.2 m, depth 0.4 m) was filled with opaque water (25 ± 1°C, depth 0.3 m) and the escape platform (10 x 10 cm) was submerged 1 cm below the surface. The swim patterns were monitored by the PC-based video-tracking system from Ethovision (Noldus Inc., Netherlands). The escape latency, swim speed, path length, and swimming paths were recorded. Mice were trained to swim to a clearly "visible platform" that was marked with a 15 cm high black flag during the first 2 d. The position of the visible platform was changed pseudo-randomly (non-spatial learning). Extra-maze cues were hidden during these trials. After 2 d of "visible platform" training, the "hidden platform" training (spatial training) was performed for 8 d. The mice had to find a hidden platform (the flag was removed) that was located at the centre of one of the four quadrants of the pool. The location of the platform was fixed throughout the running test. Mice had to navigate using extra-maze cues that were placed on the walls. Every day they received four trials with an inter-trial interval of 5 min. The mice were placed into the pool facing the sidewall randomly at one of four start locations and allowed to swim until they found the platform, or for a maximum of 90 s. Mice that failed to find the platform within 90 s were guided to the platform. The animal then remained on the platform for 20 s before being removed from the pool. After completion of the "hidden platform" training, a trial without platform was done to assess the spatial memory. For this, mice were allowed to swim freely for 90 s. The percentage of time spent in each quadrant of the pool and the number of times crossing the former position of the platform were recorded.

### Barnes maze spatial learning

A cohort of 15 *EGFL7 fl/fl;EIIa-Cre* and 15 *EGFL7 fl/fl* female mice were used for analysis of spatial learning in a Barnes maze (TSE, Bad Homburg, Germany). The animals had no prior experience of learning tasks and were 27-35 weeks old at the onset of adaptation. A classical avoidance-based test and a reward-based two-choice paradigm were employed sequentially with an interval of 35 weeks in between, the latter with a subgroup of nine and eight animals per group. The maze was divided into center, target (i.e., escape/rewarding box), opposite and neutral zones. The protocols consisted in three phases: habituation, target acquisition, and reversal or extinction learning. During habituation, mice were placed in the middle of the maze under a transparent cylinder for 20 s, and were then guided to the target hole, where they were allowed to enter the shelter. If they did not escape within 3 min, they were gently nudged into the box and allowed to stay there for 1 min. For the two-choice Barnes maze, mice were put on a mild restriction diet to increase appetite for the reward, which was placed in one of two boxes in the same quarter, two holes apart, while the other was empty. The habituation was performed on 3-4 consecutive days with two trials per day. In the target acquisition phase (3-4 d, 1 trial per day) mice were placed in the middle of the maze under an opaque cylinder for 20 s and then allowed to freely explore the maze for 5 min to find and enter the escape/reward box. In the subsequent classical REVERSAL learning (4 d, 1 trial per day), the escape box was moved to the opposite side of the maze. In the two-choice reward extinction period, only the reward was removed but positions of the boxes were maintained. EthoVision XT 11.5 (Noldus, Wageningen, Netherlands) was used for video tracking and analysis. Trial duration, distance moved, velocity, zone visits and cumulative duration in each zone were recorded.

### IntelliCage

The IntelliCage (TSE systems GmbH, Bad Homburg, Germany) experiments were performed with 16 female mice per genotype (*EGFL7*-/- or WT litters), which were 11-16 weeks old at the onset of the experiments; eight mice were housed per cage. The IntelliCage (8, 9, 10, 11) consists of four operant corners, each with two water bottles, sensors, light-emitting-diodes (LEDs) and doors that control access to water bottles. Mice were tagged with radio-frequency identiﬁcation (RFID)-transponders to record corner visits, nosepokes and lickings to observe the behavior in pre-designed tasks, summarized in Supplementary Table 2. Mice were adapted to the system for 3 d with free access to every corner, with all doors open, and water and food ad libitum (free adaptation, FA). FA was followed by a 6 d nosepoke adaptation period (NP), during which the doors remained closed, the ﬁrst nosepoke of the visit opened the door for 5 s and in order to drink more, the animals had to leave the corner and start a new visit.

#### Place avoidance acquisition (PAA) and extinction (PAEx)

In PAA, mice (4 per cage) had to learn to avoid one punishment corner, which was randomly assigned. The punishment consisted of an air puff (∼ 0.8 bar, 1 s), and it was coupled with red LED upon NP on the forbidden door, which remained closed. The avoidance acquisition lasted for 24 h. Upon completion, mice returned to their home cages for 1 d with water restriction overnight prior to their return to their IntelliCage for the analysis of the extinction of the avoidance behavior (PAEx). The IntelliCage was not cleaned to maintain environmental and olfactory cues. In PAEx, all doors opened upon nosepoke and no air-puff was applied. Only the red LED still reminded of the previously "punished" corner and was switched on upon visit of a previously forbidden corner.

#### Place preference learning

After a home cage interval, the mice returned to the IntelliCage, were re-adapted and were then adapted to daily drinking sessions (DS) for 15 d, which allowed access to the bottles only for 2 x 2 h per day from 11:00 AM – 01:00 PM and 04:00 – 06:00 PM (referred to as "module active times"). The restriction increased the motivation to learn and allowed for assessment of preference maintenance outside of the "module active times". The day-patterns were maintained during subsequent learning tasks. In the first "place preference learning" task, mice had to learn to prefer a specific corner, where they got access to the water reward on both sides (PPL). Correct corner visits were coupled with green LED. All four mice per cage were assigned to one corner. The PPL module was active only during the respective drinking sessions and lasted for 7 d. Subsequently, the challenge was increased by allowing bottle access only on one side in the rewarding corner (PPL1c1s). After 7 d, both corner and side were switched to the opposite side (PPL1c1s-REVERSAL). After relearning the corner for 7 d, a PPL-switch protocol followed, in which the correct corner switched back and forth between opposing corners during the morning and afternoon drinking sessions.

## Statistical assessment of behavioral experiments

Behavioral data are presented as mean ± SD or mean ± SEM, the latter for behavioral time courses, as specified in the respective figure legends. Data were analyzed with Graphpad Prism 6 or 8 and FlowR for IntelliCage experiments. Data were normally distributed, unless stated otherwise. Time course data or multifactorial data were submitted to two-way analysis of variance (ANOVA) using the factors 'time' and 'genotype'. In case of significant differences, groups were mutually compared at individual time points using post hoc t-tests and adjustment of the P value according to Šidák or without adjustment for between subject factor analyses of two groups. Asterisks in figures show significant differences between genotypes. For testing the null-hypothesis that groups were identical, AUCs were calculated using the linear trapezoidal rule and AUCs were compared per unpaired two-tailed t-tests.

In the IntelliCage, numbers of visits, nosepokes and licks show overall activity and drinking behavior. To assess time courses of success, the percentages of correct visits or nosepokes during the active module-times (as defined by the drinking sessions, 11:00 AM – 01:00 PM and 04:00 – 06:00 PM each day) were plotted versus time. The default modules, in which doors remained closed - outside the drinking sessions - were analyzed to assess the maintenance of rewarding corner preference. To assess the number of trials needed to achieve learning success, a probability test was used with the success criterion set to 0.35 for experiments with a random success of 0.25. Type 1 and type 2 errors were set to 0.05. The cumulative probability to achieve the respective criterion of success was plotted versus trial number to assess the proportion of memorizers in each group.

**Supplementary references**

1. Bicker F*, et al.* (2017) Neurovascular EGFL7 regulates adult neurogenesis in the subventricular zone and thereby affects olfactory perception. *Nat Commun* 8:15922.

2. Wingett SW & Andrews S (2018) FastQ Screen: A tool for multi-genome mapping and quality control. *F1000Res* 7:1338.

3. Mouse Genome Sequencing C*, et al.* (2002) Initial sequencing and comparative analysis of the mouse genome. *Nature* 420(6915):520-562.

4. Subramanian A*, et al.* (2005) Gene set enrichment analysis: a knowledge-based approach for interpreting genome-wide expression profiles. *Proc Natl Acad Sci U S A* 102(43):15545-15550.

5. Dobin A*, et al.* (2013) STAR: ultrafast universal RNA-seq aligner. *Bioinformatics* 29(1):15-21.

6. Love MI, Huber W, & Anders S (2014) Moderated estimation of fold change and dispersion for RNA-seq data with DESeq2. *Genome Biol* 15(12):550.

7. Morris R (1984) Developments of a water-maze procedure for studying spatial learning in the rat. *J Neurosci Methods* 11(1):47-60.

8. Albuquerque B, Haussler A, Vannoni E, Wolfer DP, & Tegeder I (2013) Learning and memory with neuropathic pain: impact of old age and progranulin deficiency. *Front Behav Neurosci* 7:174.

9. Hardt S*, et al.* (2017) Loss of synaptic zinc transport in progranulin deficient mice may contribute to progranulin-associated psychopathology and chronic pain. *Biochim Biophys Acta Mol Basis Dis* 1863(11):2727-2745.

10. Hardt S, Fischer C, Vogel A, Wilken-Schmitz A, & Tegeder I (2019) Distal infraorbital nerve injury: a model for persistent facial pain in mice. *Pain* 160(6):1431-1447.

11. Krackow S*, et al.* (2010) Consistent behavioral phenotype differences between inbred mouse strains in the IntelliCage. *Genes Brain Behav* 9(7):722-731.

**Supplementary Figure 1. EGFL7 expression**

EGFL7 expression in human (n=3, male) and mouse (n=3, male) hippocampal tissue. In both conditions NeuN+ cell were FACS sorted out of freshly extracted hippocampus and sequenced using bulk RNA-SEQ. For human samples we observe a significant two-fold (log2FC = 0.956508 +/- 0.1904112) increase.

**Supplementary Figure 2. Discrimination among qNSCs and aNSCs**

Fluorescence-activated cell sorting strategy for the discrimination among quiescent neural stem cells (qNSCs) and activated aNSCs. (**a**) GLAST^+^/CD133^+^ NSCs were (**b**) either additionally EGFR^+^ (aNSC) or EGFR^-^ (qNSC). A higher amount of aNSCs was detected in *EGFL7*-/- mice (5.56 ± 1.65% versus 2.20 ± 0.53% in wild-type control (WT). qNSCs did not significantly differ (94.44 ± 1.65% versus 97.8 ± 0.53%; n = 3; *p*= 0.0499). **(c)** RNA-sequencing of sorted qNSCs, aNSCs (n = 3) confirm a cell type specific molecular signature. The cell types can be discriminated by their RNA expression. The sorted aNSCs and qNSC differentially express specific genes, e.g., the proliferation markers which are typically dissimilar for qNSCs and aNSCs. Exemplary the proliferation marker mKi67 and Notch1 were plotted and both highly expressed in aNSCs but not in qNSCs.

**Supplementary Figure 3. Notch signaling pathway expression in HC-NSCs.**

X-fold expression of various Notch signaling pathway components detected by qRT-PCR from (**a**) d2 HC-NSCs and d5 HC-NSCs (**b**) derived from WT litters and *EGFL7*-/- HC. Statistical analysis was performed by Student’s t-test/Mann–Whitney U-test. Data are represented as mean ± S.D., n=3.

**Supplementary Figure 4. EGFL7-regulated genes identified by RNA-sequencing**

(**a, b**) Leading edge genes according to GSEA analysis (top 50 up and down) and exemplary top regulated gene sets.

**Supplementary Figure 5. Overall activity and motor functions of EGFL7 knock-out mice**

(**a**) In the Morris water maze, the latency to reach the visible platform, the proportion of time spent in the platform quarter and the swimming velocity were similar in both genotypes. Sample sizes were n = 19 for *EGFL7*-/- mice and n = 33 for WT litters. (**b**) Body weights were similar throughout experiments. Data shown as mean ± SD. Statistical analysis was performed by paired (visible platform) and unpaired Student's t-tests. (**c**) Time course data of the overall activity in the IntelliCage showing the number of visits, nosepokes and licks per day throughout the adaptation and learning tasks as indicated. The learning experiments are indicated as grey shaded areas, full descriptions can be found in Supplementary Table 2. Time course data are shown as mean ± SEM (n = 16 per genotype).

# Supplementary Table 1. Primers used for qRT-PCR

| **Gene** | **Pubmed (NM)** | **Forward (5' - 3')** | **Reverse (5' - 3')** |
| --- | --- | --- | --- |
| *Egfl7* | NM_178444.5 | cacctaccgaaccatctacc | acatggaggctggcatattg |
| *Rps13* | NM_026533.3 | ttcaccgattggctcgatac | ttatgccactagagcagagg |
| *RNAPII* | NM_027231.2 | gacaaaactggctcctctgc | gcttgccctctacattctgc |
| *VEGFD* | NM_010216.3 | tgcctgggacagaagaccact  tgcctgggacagaagaccact | actgcagttttccgggtgct  actgcagttttccgggtgct |

**Supplementary Table 2. Summary of the protocols and behavioral tasks in the IntelliCage**

| **Task name** | **Task description** | **Punishment** | **Duration** | **Drinking time** |
| --- | --- | --- | --- | --- |
| Free adaptation (FA) | Habituation to the system with free access to every corner, with all doors open, and water and food ad libitum. | No | 3 days | No restriction |
| Nosepoke adaptation (NP) | The first nosepoke of a visit opened the door for 5 s. To drink more, the animals had to leave the corner and start a new visit. | No | 6 days | No restriction |
| Place avoidance acquisition (PAA) | Mice had to avoid a specific corner. NP in this corner was punished with an air-puff and a red LED was switched on upon visit to this corner. Avoidance acquisition was for 24h, followed by a one-day home cage interval. | Airpuff | 1 day | No restriction |
| Place avoidance extinction (PAex) | During avoidance extinction, water was available in each corner on NP without any punishment. Only the red LED still announced the previously punished corner. | No | 5 days | No restriction |
| Nosepoke drinking session (DS) | To increase the learning drive, mice were adjusted to defined drinking sessions, in which the “nosepoke” task was active. The start of the drinking time was announced with green LED lights for 2 min. | No | 15 days | Restricted to 11.00AM-01:00PM and  04.00PM-06.00PM |
| Place preference learning one corner two sides (PPL) with drinking session | Mice were allowed to drink in only one corner on both sides. Visit of the correct corner was announced with green LED. Only the first correct nosepoke of a visit opened the door for 5 s. | No | 7 days | Restricted to 11.00AM-01:00PM and  04.00PM-06.00PM |
| Place preference learning one corner one side (PPL1c1s) with drinking session | Mice were allowed to drink in only one corner only on one side. LED announced the correct side. Only the first correct nosepoke of a visit opened the door for 5 s. | No | 7 days | Restricted to 11.00AM-01:00PM and  04.00PM-06.00PM |
| Place preference REVERSAL (PPL1sREV) | Protocol as in PPL1c1s but with the opposite corner / sides were correct. | No | 7 days | Restricted to 11.00AM-01:00PM and  04.00PM-06.00PM |
| Place preference REVERSAL (PPLswitch) | Protocol as in PPL1c1s but the correct corner and sides switched to the respective opposite sides between the morning and afternoon drinking sessions. | No | 7 days | Restricted to 11.00AM-02:00PM and  11.00PM-02.00AM |
