# Supplementary figures and images for: EGFL7 loss correlates with increased VEGF-D expression, upregulating hippocampal adult neurogenesis and improving spatial learning and memory
Source: Cell Mol Life Sci. 2023 Jan 30;80(2):54. doi: 10.1007/s00018-023-04685-z (PMC9886625; doi:10.1007/s00018-023-04685-z)

Suppl. Figure 1

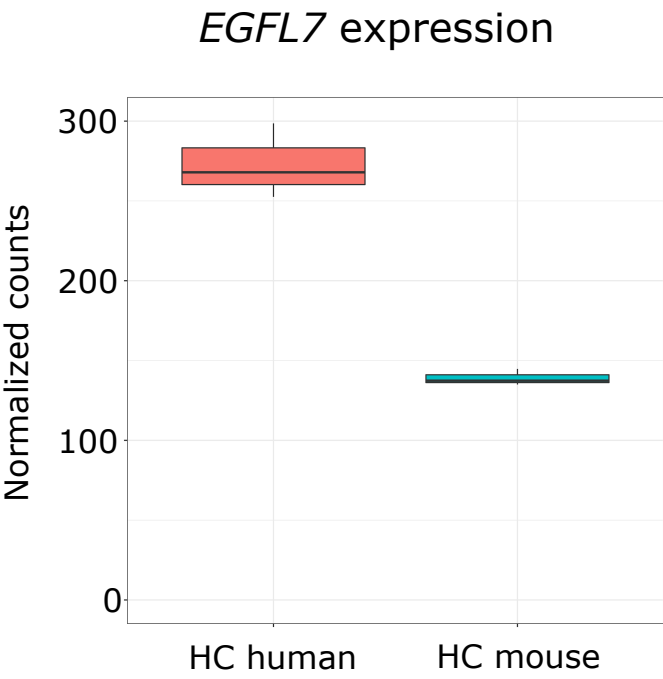

Supplement: Supplementary file 2 — Supplementary file2 (PDF 66 KB) [file 18_2023_4685_MOESM2_ESM.pdf]

Suppl. Figure 2

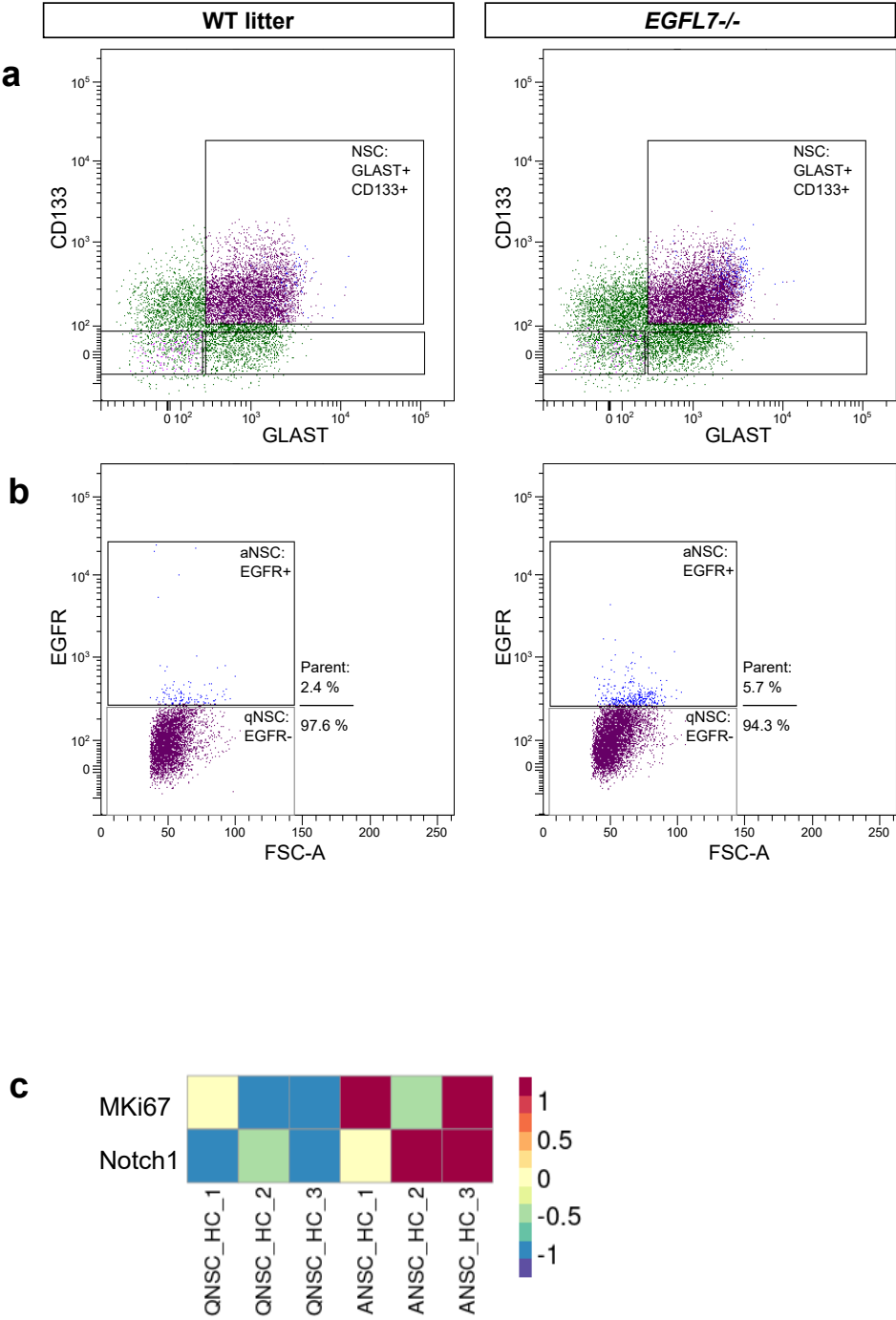

Supplement: Supplementary file 3 — Supplementary file3 (PDF 438 KB) [file 18_2023_4685_MOESM3_ESM.pdf]

Suppl. Figure 3

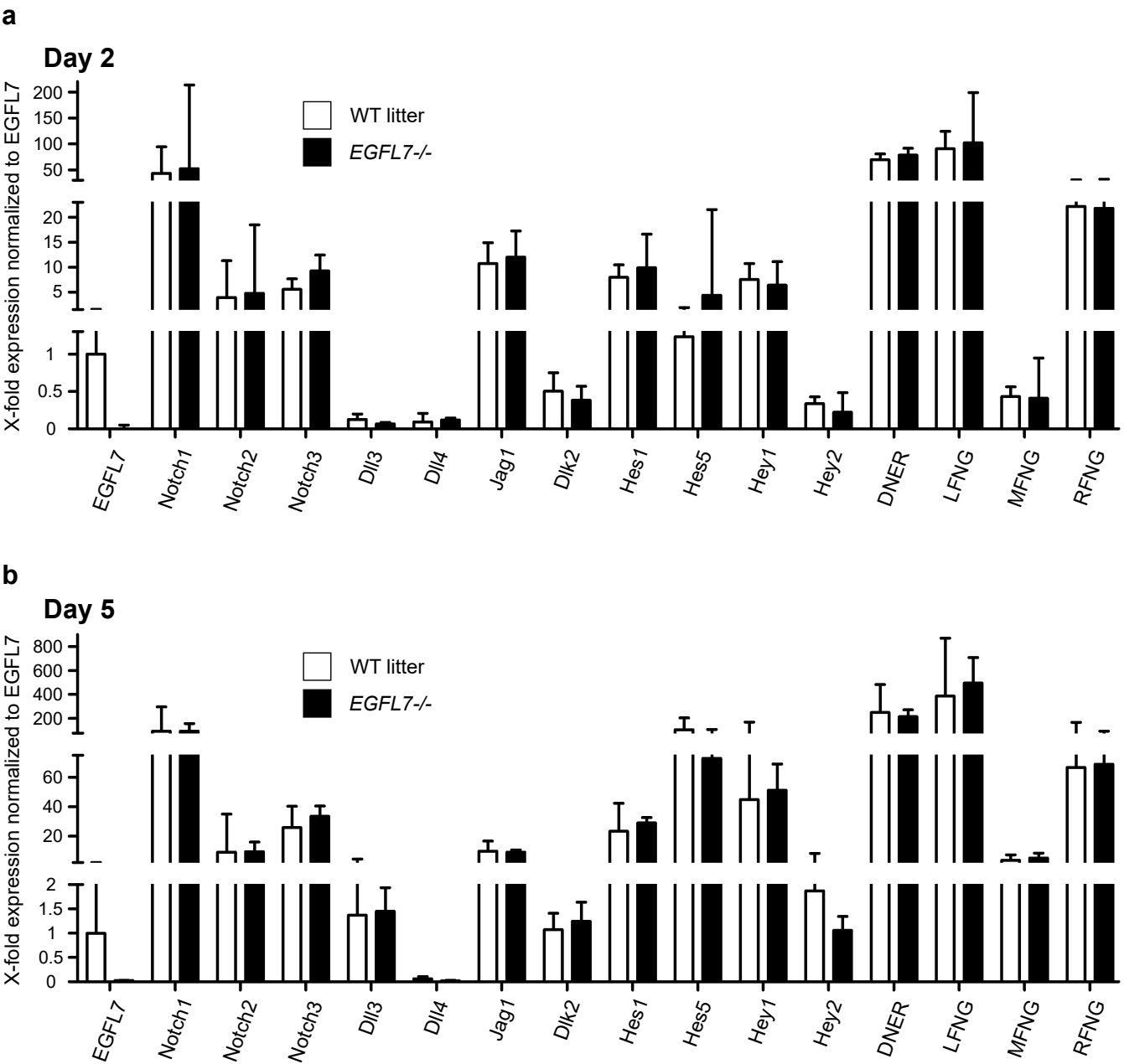

Supplement: Supplementary file 4 — Supplementary file4 (PDF 58 KB) [file 18_2023_4685_MOESM4_ESM.pdf]

**a**

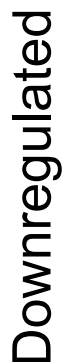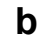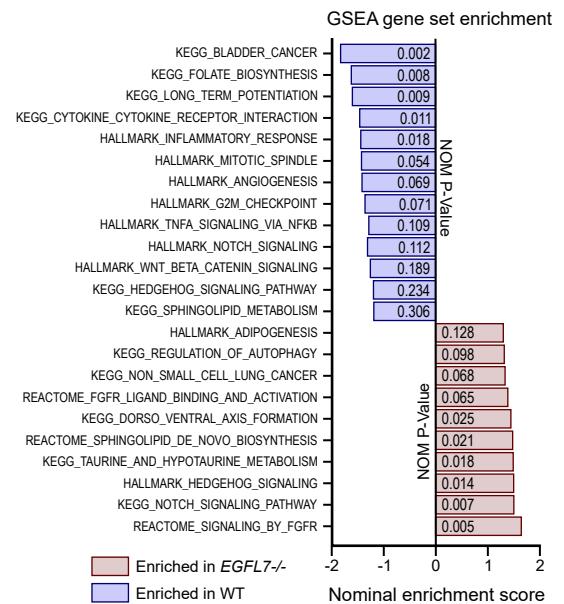

Supplement: Supplementary file 5 — Supplementary file5 (PDF 206 KB) [file 18_2023_4685_MOESM5_ESM.pdf]

Suppl. Figure 5

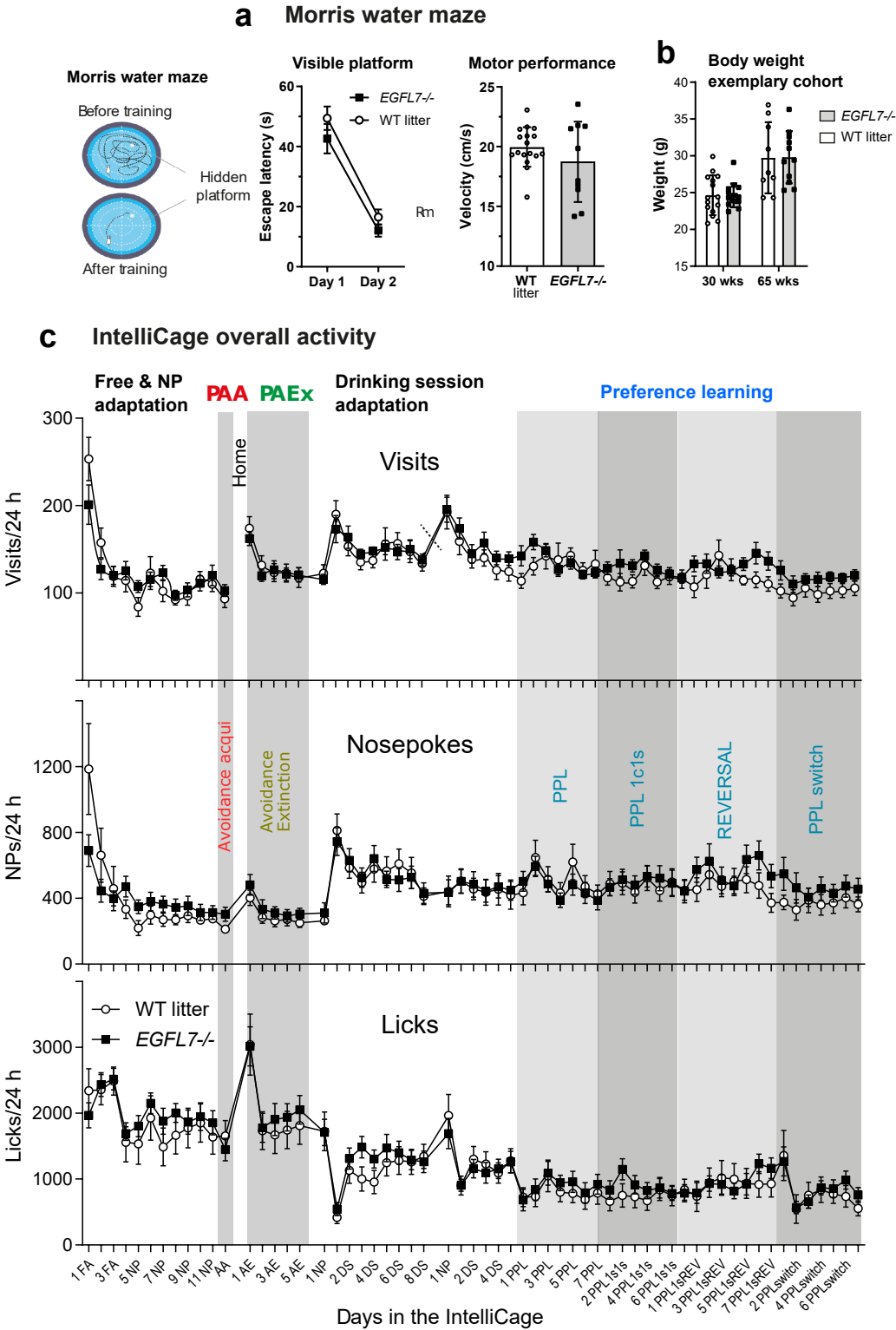

Supplement: Supplementary file 6 — Supplementary file6 (PDF 143 KB) [file 18_2023_4685_MOESM6_ESM.pdf]
